# Recent advancement on photocatalytic plastic upcycling

**DOI:** 10.1039/d3sc05555h

**Published:** 2023-12-27

**Authors:** Jingrun Ran, Amin Talebian-Kiakalaieh, Shuai Zhang, Elhussein M. Hashem, Meijun Guo, Shi-Zhang Qiao

**Affiliations:** a School of Chemical Engineering, University of Adelaide Adelaide SA 5005 Australia s.qiao@adelaide.edu.au

## Abstract

More than 8 billion tons of plastics have been generated since 1950. About 80% of these plastics have been dumped in landfills or went into natural environments, resulting in ever-worsening contamination. Among various strategies for waste plastics processing (*e.g.*, incineration, mechanical recycling, thermochemical conversion and electrocatalytic/photocatalytic techniques), photocatalysis stands out as a cost-effective, environmentally benign and clean technique to upcycle plastic waste at ambient temperature and pressure using solar light. The mild reaction conditions for photocatalysis enable the highly selective conversion of plastic waste into targeted value-added chemicals/fuels. Here, we for the first time summarize the recent development of photocatalytic plastic upcycling based on the chemical composition of photocatalysts (*e.g.*, metal oxides, metal sulfides, non-metals and composites). The pros and cons of various photocatalysts have been critically discussed and summarized. At last, the future challenges and opportunities in this area are presented in this review.

## Abstract 2

This review critically summarizes the recent achievement on various photocatalysts for plastic upcycling. A range of key factors affecting the reaction kinetics/thermodynamics are also summarized.

## Graphical abstract

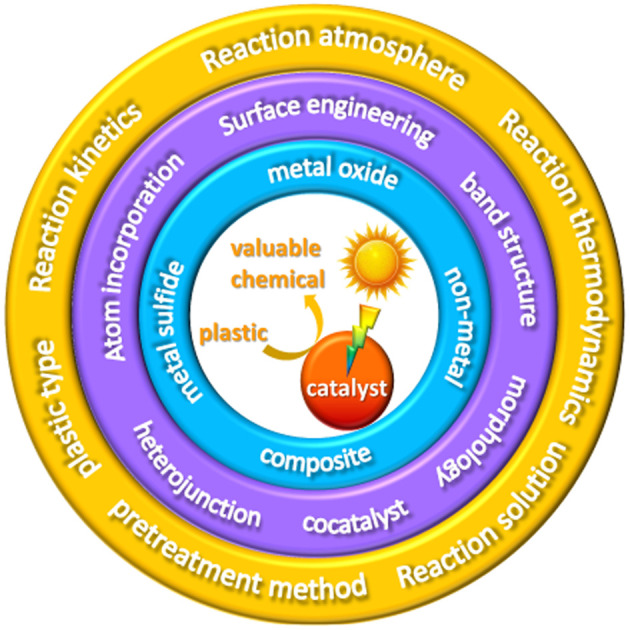

## 1. Introduction

Up till now, more than 8 billion tons of plastics have been synthesized, in which less than 20% of them are incinerated or recycled. Around 80% of used plastics are accumulated in the natural environment or ended up in landfills.^1–33^ Recently, various dealing strategies have been developed for treating plastic wastes, such as incineration, mechanical recycling, thermochemical conversion and electrocatalytic/photocatalytic techniques.^1–33^ Among them, large-scale incineration and landfills are the two most general routes because they are inexpensive, facile and adaptable to various feedstock.^1–33^ Unfortunately, these two traditional routes are susceptible to deleterious environmental impacts and negligible added values.^1–33^ Low value products are manufactured by mechanical recycling, which down-cycles plastic wastes into low value products and is also restricted to single-component and few types of clean thermoplastics.^1–33^ In contrast, upcycling conversion of plastic wastes, *e.g.*, thermocatalytic/photocatalytic/electrocatalytic techniques, has attracted significant attention recently, since value-added chemicals/fuels or materials with extra economic value can be acquired *via* these appealing techniques.^1–33^ Among these techniques, photocatalysis stands out as a cost-effective, environmentally-benign and green strategy able to upcycle plastic wastes at ambient temperature and pressure utilizing renewable sunlight.^34–79^ A photocatalysis reaction conducted in mild conditions is anticipated to accurately activate target chemical bonds, while reserving the other functional groups, thus realizing high selectivity to desirable products.^34–49^

Recently, various photocatalysts, such as metal oxides (*e.g.*, Pt loaded P25 TiO_2_ (ref. 34 and 35) and Co doped Ga_2_O_3_ (ref. 37)), metal sulphides (*e.g.*, MoS_2_ tipped CdS nanorods^39^), non-metals (*e.g.*, graphitic carbon nitride)^41–44^ and composites (*e.g.*, ZnO encapsulated in UiO66-NH_2_ (ref. 46) and Ag_2_O encapsulated in Fe based MOFs^47^), have been developed for catalytic upcycling of plastics. To date, many reviews have covered upcycling of plastic waste,^1–31,80,81^ including various upcycling techniques such as the photo-electrochemical (PEC) technique, photo-thermal technique, photocatalysis technique, thermo-catalysis technique, bio-catalysis technique, pyrolysis technique, hydrogenolysis technique, solvolysis technique, hydrolysis technique, microwave-assisted technique, plasma-assisted technique, mechanical technique and combination of these techniques.^3,5–17,19–33,81^ Among these reviews, the introduction/discussion of the photocatalysis technique is not insightful and comprehensive. Some reviews only introduce the mechanisms and applications of various photocatalysts for plastic upcycling.^1,2,4,6,18,80^ But the advantages/disadvantages of various photocatalysts are rarely discussed and highlighted. Besides, the structure–performance relationship of these photocatalysts is much less summarized. Moreover, the insightful reaction mechanism of photocatalytic plastic upcycling under various reactions conditions is still not very clear at this stage. Especially, oxygen plays a key role in plastic upcycling, which should be explained more clearly and thoroughly.

In this review, we for the first time summarize and review recently reported photocatalysts for plastic upcycling based on their chemical compositions including metal oxides, metal sulphides, non-metals and composites. The accurate advantages/disadvantages of photocatalysts are critically analysed and discussed according to their chemical compositions. Especially, the perceptive reaction mechanisms for various photocatalysts under varied reaction conditions are also introduced and summarized in this review.

## 2. Fundamentals of photocatalytic plastic upcycling

Owing to the ultrastability of most plastics, such as polyolefins and polyesters, it is very challenging to directly convert these plastics into valuable chemicals *via* photocatalysis at ambient temperature and pressure using sunlight only. Thus, in most cases, these plastics are usually pre-treated by various routes to release monomers/oligomers or to be converted into short-chain carbon-based molecules before photocatalysis treatment.^34,38–43,49^ For example, polyesters, *e.g.*, polyethylene terephthalate (PET), polylactic acid (PLA) and polyurethane (PUR), are usually pre-treated in alkaline solution at slightly raised temperature (*e.g.*, 40–70 °C) to be hydrolysed, thus releasing abundant monomers/oligomers.^38–43,49^ Additionally, polyolefins, *e.g.*, polyethylene (PE), are usually hydrothermally treated in nitric acid aqueous solution, to be transformed into various short-chain carboxylic acids, *e.g.*, succinic acid, glutaric acid, acetic acid, adipic acid and propanoic acid.^34,39^ On the other hand, in some rare cases, these plastics, *e.g.*, PE, polyvinyl chloride (PVC), polypropylene (PP) and polystyrene (PS), are directly added into the reaction system for photocatalytic plastic upcycling, which can yield value-added chemicals/fuels, *e.g.*, H_2_, CO, formic acid, acetic acid, benzoic acid, acetophenone and benzaldehyde.^36,37,44–46,48^ In some of these cases, metal oxide photocatalysts with strongly oxidative photo-induced holes are applied.^36,37,46^ In the other case, an air/O_2_ atmosphere and raised temperature/pressure are adopted to boost the oxidative cleavage of the strong C–C/C–H bonds in these untreated plastics.^44^ Another key reaction condition for photocatalytic plastic upcycling is the existence/absence of oxygen (O_2_) in the reaction system. If photocatalytic plastic upcycling is conducted under aerobic conditions, abundant reactive oxygen species (ROS), such as ˙O_2_^−^, ˙HO_2_^−^ and ˙OH radicals, will be produced by the following reactions:^36,37,44,46–48^

1 e^−^ + O_2_ → ˙O_2_^−^

2 ˙O_2_^−^ + e^−^ + 2H^+^ → H_2_O_2_

3 H_2_O_2_ + *hν* → 2˙OH

4 H_2_O_2_ + ˙O_2_^−^ → ˙OH + O_2_ + OH^−^

5 e^−^ + H_2_O_2_ → ˙OH + OH^−^

These ROSs will significantly boost the oxidative cleavage of the C–C/C–H/C–O/C–N/C–Cl bonds in the robust plastics and the production of short-chain carbon-based molecules as valuable chemicals/fuels.^36,37,44,46–48^ But O_2_ in the reaction system can compete with H^+^/H_2_O to obtain photo-induced electrons, resulting in a much lower H_2_ yield under aerobic conditions compared to that under anaerobic conditions. In contrast, if photocatalytic plastic upcycling is performed under anaerobic conditions, these ROSs are much more challenging to form in the reaction system unless the photo-induced holes with sufficient oxidation ability can oxidize OH^−^/H_2_O to yield ˙OH radicals. Without the competition from O_2_, many more photo-excited electrons can be utilized for H_2_ evolution, resulting in usually a much higher H_2_ evolution rate under anaerobic conditions compared to that under aerobic conditions.

**Fig. 1:**
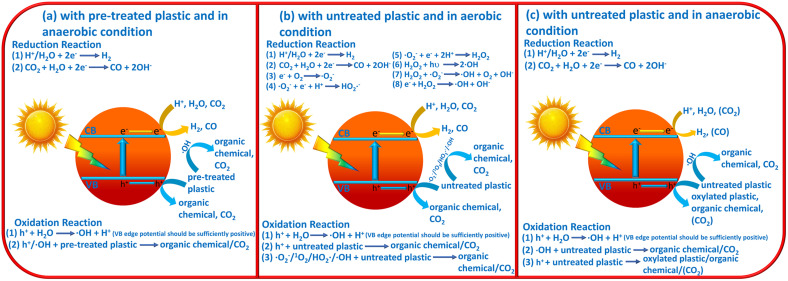
Schematic images for (a) photocatalytic plastic upcycling using pre-treated plastics and under anaerobic conditions, (b) photocatalytic plastic upcycling using untreated plastics and under aerobic conditions and (c) photocatalytic plastic upcycling using untreated plastics and under anaerobic conditions.

On the basis of the above introductions, the reaction mechanisms for photocatalytic plastic upcycling are categorized into three different types: (i) photocatalytic upcycling with pre-treated plastics and under anaerobic conditions;^34,38–43,49^ (ii) photocatalytic upcycling with untreated plastics and under aerobic conditions;^36,37,44,46,48^ (iii) photocatalytic upcycling with untreated plastics and under anaerobic conditions^45^ (Fig. 1a–c). Besides, we have also discussed the reaction thermodynamics and kinetics for plastic upcycling as well as engineering routes on photocatalysts.

### 2.1 Plastic upcycling with pre-treated plastics and under anaerobic conditions

As the most reported reaction condition for plastic upcycling,^34,38–43,49^ the reaction mechanism is summarized in Fig. 1a: (i) monomers/oligomers/short-chain carbon-based molecules/surface oxygenated plastics are first generated from the pre-treatment of various plastics; (ii) under anaerobic conditions, photo-excited electrons mainly involve in H_2_ evolution, thus resulting in a relatively high photocatalytic H_2_ evolution rate under these reaction conditions; (iii) photo-induced holes can oxidize the formed monomers/oligomers/short-chain carbon-based molecules to yield value-added chemicals/fuels; (iv) in some cases, if metal oxide photocatalysts with strongly oxidative holes are utilized, these monomers/oligomers/short-chain carbon-based molecules could be over-oxidized to yield CO_2_, which could be further reduced by photo-generated electrons to form CO.

### 2.2 Plastic upcycling with untreated plastics and under aerobic conditions

Another often-reported reaction condition is plastic upcycling with untreated plastics and under aerobic conditions. The reaction mechanism is shown in Fig. 1b: (i) plastic powder is directly added into the reaction system; (ii) photocatalysts with strongly oxidative photo-induced holes, *e.g.*, Nb_2_O_5_ atomic layers,^36^ Co doped Ga_2_O_3_ (ref. 37) and ZnO coupled UiO66-NH_2_,^46^ are usually adopted in this reaction system, which can oxidize H_2_O/OH^−^ to form highly oxidative ˙OH radicals; (iii) the photo-induced electrons can involve in the reaction with O_2_ to form various ROSs, *e.g.*, ˙O_2_^−^, ^1^O_2_, ˙HO_2_^−^ and ˙OH; (iv) all the formed ROSs and strongly oxidative holes can oxygenate the plastics to yield hydroxyl, carbonyl and carboxyl groups, thus polarizing and greatly weakening the inert C–C/C–O/C–H/C–N bonds in plastics; (v) these C–C/C–O/C–H/C–N bonds of oxygenated plastics can be further oxidatively cleaved to yield short-chain carbon-based molecules or even over-oxidized to form CO_2_; (vi) photo-induced electrons can reduce the formed CO_2_ to yield CO or even carbon-based molecules, *e.g.*, acetic acid;^36^ (vii) photo-induced electrons can also reduce H^+^/H_2_O to yield H_2_; (viii) in some cases, non-metal photocatalysts with moderate oxidation ability, *e.g.*, g-C_3_N_4_, and organic solvent,^44^*e.g.*, acetonitrile, instead of aqueous solution, are applied in the reaction system with raised temperature (*e.g.*, 70 °C) rather than room temperature (25 °C).

### 2.3 Plastic upcycling with untreated plastic and under anaerobic conditions

Photocatalytic plastic upcycling under these reaction conditions is rarely reported.^45^ And the reaction mechanism is not very clear. So we propose a possible mechanism in Fig. 1c as follows: (i) for the reduction reaction, under anaerobic conditions, photo-excited electrons mainly involve in H_2_ evolution *via* reducing H^+^/H_2_O in the reaction system. In some cases, CO_2_ could be generated due to the overoxidation of untreated plastic by strongly oxidative ˙OH and holes. Thus, generated CO_2_ can be further reduced by photo-excited electrons to produce some carbon-based fuels/chemicals; (ii) for the oxidation reaction, the photo-induced holes with sufficient oxidation ability can react with the H_2_O molecule to generate strongly oxidative ˙OH. Thus, ˙OH can react with untreated plastics to generate organic chemicals and CO_2_. In contrast, photo-excited holes with weak oxidative capacity can hardly cleave the C–C bond in untreated plastics to produce organic chemicals. And no CO_2_ would be generated under these conditions. Overall, photocatalytic plastic upcycling under these reaction conditions is rarely reported and more studies under these reaction conditions should be conducted to understand the reaction mechanism.

### 2.4 Reaction thermodynamics and kinetics for plastic upcycling

Reaction thermodynamics and kinetics for photocatalytic upcycling of plastic wastes into fuels/valuable chemicals are complex. Nevertheless, compared to photocatalytic water splitting with a theoretical thermodynamic requirement of 1.23 eV (Δ*G*^0^ = +237 kJ mol^−1^), the thermodynamic requirement for photocatalytic plastic upcycling is usually much lower. This is because the oxidation of plastics, especially for small molecules from pre-treated plastics, is much more facile than water oxidation for O_2_ evolution. For instance, reforming ethylene glycol and lactic acid, from pre-treated PET and PLA, shows the Gibbs free energy changes of +9.2 and +27 kJ mol^−1^, respectively. These are much lower than that of water splitting (*G*^0^ = +237 kJ mol^−1^). Thus, the much less thermodynamics requirement for plastic oxidation compared to water splitting enables the utilization of a semiconductor photocatalyst with a smaller band gap, which exhibits a much broader light absorption range for the solar spectrum. Thus, nitride, sulphide, phosphate, arsenide, selenide and even tellurite catalysts, which show deficient valence band potential for water oxidation, could be suitable for photocatalytic plastic oxidation, especially for small molecules from pre-treated plastics. Thus, abundant and undesirable plastic wastes serve as an efficient hole scavenger to increase the electron–hole separation/transfer efficiency, thus boosting the reduction reaction (H_2_ evolution or CO_2_ reduction). Simultaneously, value-added chemicals/fuels can be generated *via* plastic oxidation.

Reaction kinetics in photocatalytic plastic upcycling are very complex and are affected by many factors, such as plastic type, pre-treatment route, reaction solution and reaction atmosphere. First, the types of plastics obviously affect the reaction kinetics. Polyolefins (*e.g.*, PE, PP, PS and PVC), which account for 57% of the total plastics, consist of inert C–C and C–H bonds with high dissociation energies. Thus, reaction kinetics for breaking these C–C/C–H bonds and upcycling polyolefins are very sluggish. Besides, the hydrophobic nature of polyolefins makes their dispersion in aqueous solution and adsorption on photocatalysts very challenging. In contrast, polyesters/polyamides (*e.g.*, PET, PLA, PUR) with ester/amide bonds are much easier to decompose to yield the corresponding monomers, which are facilely adsorbed on photocatalysts for further forming value-added chemicals.

Second, the pre-treatment route also greatly affects the reaction kinetics. Without pre-treatment, the reaction kinetics for upcycling most plastics are rather slow, especially for inert polyolefins. The hydrothermal pre-treatment of polyolefins (*e.g.*, PE) in nitric acid could cleave the C–C bond and convert most of them into various carboxylic acids (*e.g.*, succinic acid, glutaric acid, acetic acid, adipic acid and propanoic acid). These short-chain water-soluble carboxylic acids could be more easily adsorbed onto photocatalysts and further yield value-added chemicals/fuels more efficiently. Furthermore, the hydrolysis pre-treatment of polyesters/polyamides (*e.g.*, PET and PUR) in alkaline solution at elevated temperature could yield the corresponding monomers, which can be easily dissolved in aqueous solution and adsorbed on photocatalysts for more efficient conversion into valuable chemicals/fuels. Moreover, the plasma treatment of plastics at room temperature and atmosphere could graft oxygen-containing functionalities on the PE/PP/PVC surface, leading to increased hydrophilicity in water and more intimate interaction with photocatalysts. Thus, reaction kinetics for yielding fuels/valuable chemicals from pre-treated PE/PP/PVC is improved to some extent. Nevertheless, since this plasma treatment doesn't convert PE/PP/PVC into short-chain monomers/oligomers, the enhancement of reaction kinetics for plastic upcycling is limited.

Third, different reaction solutions (*e.g.*, pure water, alkaline aqueous solution and organic solvent) also obviously affect the reaction kinetics for plastic upcycling. As pure water is applied as the reaction solution, the reaction kinetics for upcycling most plastics in pure water are very slow. This is because that most of the plastics are hydrophobic, which float on the surface of pure water or precipitate at the bottom rather than suspend in the water, making their adsorption or interaction with photocatalysts challenging. Besides, the undissolved plastics also hinder light absorption by photocatalysts to some extent. In contrast, as alkaline aqueous solution (*e.g.*, NaOH and KOH) is applied as the reaction solution, some kinds of plastics, such as polyesters/polyamides, could be dissolved/hydrolysed in alkaline aqueous solution, yielding monomers easily adsorbed on photocatalysts for upcycling reactions. Thus, the corresponding reaction kinetics for these plastics are obviously improved. Nevertheless, this alkaline environment could corrode the photocatalysts and reduce their activity/selectivity/stability. As organic solvents (*e.g.*, acetonitrile, tetrahydrofuran, cyclohexane and toluene) are utilized as the reaction solution, hydrophobic plastics (*e.g.*, PE, PP, PS and PVC) can be dissolved in these organic solvents with stirring and elevated temperature. These will boost their adsorption on photocatalysts and further transformation into valuable chemicals/fuels. The activity/selectivity/stability of photocatalysts will also be affected. Moreover, by-products from these organic solvents might also be generated.

Fourth, the reaction atmosphere considerably influences the reaction kinetics for plastic upcycling. Under aerobic conditions, various ROS species (*e.g.*, ˙O_2_^−^, ^1^O_2_, ˙HO_2_^−^, ˙OH and H_2_O_2_) could be generated to greatly boost the oxidation and conversion of plastics into value-added chemicals/fuels. Nevertheless, strongly oxidative and non-selective ROS could over-oxidize these plastics and generate CO_2_. In contrast, under anaerobic conditions, only photo-excited holes (and ˙OH) involve in the oxidation and conversion of plastics into valuable chemicals/fuels. Thus, it is much easier to regulate the oxidation ability of photo-excited holes for controlling the selectivity of plastic upcycling. Nevertheless, due to the lack of massive ROS species for oxidation of plastics, the reaction kinetics of plastic upcycling is reduced.

### 2.5 Engineering routes on photocatalysts

A range of engineering routes have been developed for optimizing the physicochemical features of photocatalysts to achieve increased photocatalytic activity/selectivity/stability for plastic upcycling. For instance, researchers have reported various strategies, such as loading co-catalysts,^34,35,39–43,45^ doping engineering,^37^ morphology engineering,^36^ surface engineering,^38^ band structure engineering^40^ and forming heterojunctions,^45–49^ which can significantly increase the photocatalytic performances. The accurate structure–performance relationship and reaction mechanism for these photocatalysts will be introduced in Section 3 as follows.

## 3. Photocatalysts for plastic upcycling

Currently, the reported photocatalysts for plastic upcycling are categorized into metal oxide based photocatalysts,^34–37^ metal sulphide based photocatalysts,^38–40^ non-metal based photocatalysts^41–44^ and composite photocatalysts.^45–49^ We will introduce the research in these fields according to the above four categories.

### 3.1 Metal oxide based photocatalysts for plastic upcycling

Various metal oxides, such as TiO_2_,^34,35^ Nb_2_O_5_ (ref. 36) and Ga_2_O_3_,^37^ have been applied for photocatalytic plastic upcycling. Owing to the strong oxidation abilities of metal oxide based photocatalysts arising from their deep O 2p derived valence band (VB), they are often adopted to upcycle polyolefins with strong C–C bonds, such as high density polyethylene (HDPE), low density polyethylene (LDPE), polypropylene (PP) and polyvinyl chloride (PVC). With light irradiation, they can generate strongly oxidative photo-induced holes and yield exceptionally oxidative hydroxyl radicals (˙OH) in aqueous solution under aerobic/anaerobic conditions (h^+^ + OH^−^ → ˙OH). If applied in an air/O_2_ atmosphere, the photo-induced electrons in the CB of these metal oxide based photocatalysts usually can react with O_2_ to form a series of oxidative reactive oxygen species (ROSs), such as ˙O_2_^−^ and ˙HO_2_^−^ radicals. These ROSs (*e.g.*, ˙OH, ˙O_2_^−^ and ˙HO_2_^−^) can first oxidize polyolefins to form hydroxyl, carbonyl and carboxyl functionalities. Then, the strong C–C/C–H bonds of these polyolefins will be polarized and significantly weakened, resulting in the much easier cleaving of these C–C/C–H bonds and upcycling of these polyolefins into value-added chemicals/fuels.

**Fig. 2:**
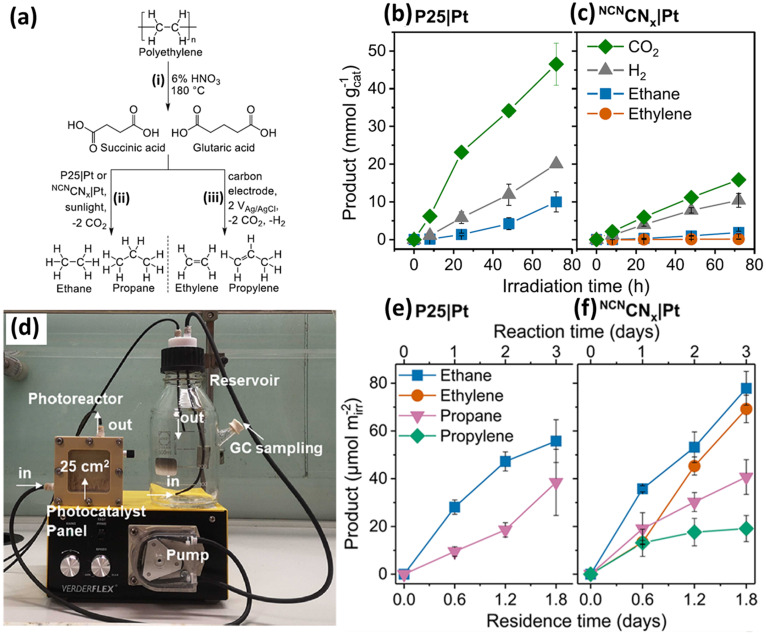
(a) Hydrothermal pre-treatment of PE to form dicarboxylic acid (i) followed by conversion into gaseous hydrocarbon using photocatalysis to yield alkanes (ii) or electrolysis to yield alkenes (iii). (b) Photocatalytic reforming of succinic acid in 0.1 M HNO_3_ using (b) P25|Pt or (c) ^NCN^CN_*x*_|Pt. Reaction conditions: AM 1.5G (100 mW cm^−2^), 25 °C, 2 ml of 10 mg ml^−1^ succinic acid in 0.1 M HNO_3_ (pH set to be 4), and 2 mg ml^−1^ photocatalyst. (d) Image of the photocatalytic flow setup. The pre-treated PE solution (not displayed in this image) in the reservoir is continuously pumped with a peristaltic pump into the photoreactor with an irradiation area of 25 cm^2^. Then, the solution is pumped into the reservoir again; the generated gaseous products are sampled and studied by gas chromatography. Photocatalytic product generation using a flow setup with (e) P25|Pt and (f) NCNCN_*x*_|Pt. Reproduced with permission from copyright 2021, American Chemical Society.^34^

First, we will introduce two studies using the most extensively studied metal oxide photocatalyst, TiO_2_, for photocatalytic plastic upcycling under anaerobic conditions with pre-treated polyolefins.^34,35^ Both these studies adopt commercial P25 TiO_2_ loaded with the Pt cocatalyst. The Reisner^34^ group first used 6% nitric acid and a hydrothermal reaction at 180 °C for 4 hours to convert ∼40% polyethylene (PE) into a variety of liquid chemicals including succinic acid (44%), glutaric acid (22%), acetic acid (21%), adipic (9%) and propanoic acid (4%). Succinic acid and glutaric acid are identified as the major products from PE conversion (Fig. 2a). Then, they synthesized 1 wt% Pt nanoparticle (NP) loaded P25 TiO_2_ using a chemical reduction route. After a 96 hour reaction using the PE decomposition solutions, the as-synthesized 1 wt% Pt NP loaded P25 TiO_2_ exhibits photocatalytic performance for the evolution of ethylene (0.017 mmol g^−1^), ethane (0.25 mmol g^−1^), propylene (0.007 mmol g^−1^), propane (0.14 mmol g^−1^), H_2_ (6.3 mmol g^−1^) and CO_2_ (5.9 mmol g^−1^). Thus, ethane and propane are detected as the major alkane products from photo-reforming of PE decomposition solution (Fig. 2a). They found that only a small amount of ethylene is generated in this reaction, attributed to the efficient transfer of adsorbed hydrogen to the intermediate radical on 1 wt% Pt NP loaded P25 TiO_2_. Some of the generated CO_2_ and H_2_ arise from the decarboxylation reaction. Besides, due to the strong oxidation abilities of photogenerated holes in TiO_2_, mineralization of the acquired chemicals (*e.g.*, succinic and glutaric acid) after pre-treatment can occur, also leading to CO_2_ and H_2_ evolution. ^13^C-labelled succinic acid using 1 wt% Pt NP loaded TiO_2_*via*^1^H-nuclear magnetic resonance (^1^H NMR) spectroscopy confirms that the evolved ethane is generated from the succinic acid. They found that without Pt as the co-catalyst, larger amounts of ethylene and smaller amounts of ethane are observed. Ethane became the major product again as MoS_2_ was adopted as the co-catalyst. They have also synthesized Pt loaded cyanamide-regulated carbon nitride powder (^NCN^CN_*x*_|Pt). After a 72 hour photocatalytic reaction, the major alkane products from P25|Pt and ^NCN^CN_*x*_|Pt are ethane at 56.3 and 7.2 μmol g^−1^ h^−1^ (Fig. 2b and c). Then, they have set up a flow photocatalytic reactor system (Fig. 2d), in which a continuing generation of ethane and propane was achieved for both P25|Pt and ^NCN^CN_*x*_|Pt (Fig. 2e and f), together with the constant production of ethylene and propylene for ^NCN^CN_*x*_|Pt (Fig. 2f). In another study, a plasma pre-treatment strategy was reported to treat polyolefins to partially cleave the C–C/C–H bonds and generate oxygenated functional groups on the backbones of PE, PP or PVC.^35^ FTIR and XPS spectra together confirm the formation of hydroxyl, carboxyl and carbonyl functionalities on the surface of PE. Contact angle measurement further reveals the gradually reduced contact angle of PE with water as plasma treatment time increases, suggesting the increased hydrophilicity of treated PE. This will lead to better dispersion of treated PE in aqueous solution and more intimate contact between the photocatalyst and treated PE. Molecular dynamics computations reveal a stronger interaction between the TiO_2_ surface and plasma treated PE compared to that between the TiO_2_ surface and untreated PE.

Pt NPs with sizes of 5–15 nm are loaded on P25 TiO_2_*via* photo-deposition to synthesize a Pt–TiO_2_ photocatalyst. Photocatalytic experiments show that H_2_ evolution first rises and then decreases with increasing plasma treatment time on PE using Pt–TiO_2_. This is because the first activity increase arises from the generation of abundant –OH functionalities on the surface of PE. The subsequent activity decrease is due to the formation of massive carbonyl and carboxyl functionalities on treated PE. Reforming of PE also generates CH_4_, C_2_H_4_ and C_2_H_6_. But no liquid products were found by ^1^H NMR for short-/long-term photocatalysis tests. This plasma treatment effect also increased the H_2_ evolution activity on treated PP or PVC compared to untreated PP or PVC, suggesting the universality of this route. When comparing the above two studies using the same photocatalyst (Pt loaded P25 TiO_2_) under similar reaction conditions (Table 1), we can find that the H_2_/C_2_H_4_/C_2_H_6_/CO_2_ evolution rates of PE upcycling using the hydrothermal pre-treatment are much higher than those of PE upcycling using the plasma pre-treatment. These are easy to understand because the hydrothermal treatment has already converted most of the PE into C_2−6_ based carboxylic acids, which are much easier to upcycle into short-chain molecule chemicals/fuels. In comparison, plasma treatment only oxygenated PE with –OH, C=O and O–C=O functionalities without effectively cleaving the backbones of PE to form small molecules. Nevertheless, the hydrothermal pre-treatment using nitric acid is energy-intensive with potential environmental concerns. Therefore, it is desirable to develop an efficient strategy for photocatalytic upcycling of polyolefins with cost-effective and environmentally benign pre-treatment routes or even without pre-treatment.

**Table 1:** Metal oxide based photocatalysts for photo-reforming of plastics

Metal oxide based catalyst	Pre-treatment	Product after pre-treatment	Reaction conditions	Activity and stability	Reference (year)
1 wt% Pt loaded P25 TiO_2_	180 °C, 4 h hydrothermal reaction using 6% HNO_3_ aqueous solution	PE converted to succinic acid (44%), glutaric acid (22%), acetic acid (21%) adipic acid (9%), propanoic acid (4%)	Solar simulator (AM 1.5G, 100 mW cm^−2^) with a water filter (removing infrared light), 0.2 ml PE decomposition solution and 1.8 ml H_2_O, pH = 4, 4 mg catalyst, N_2_ atmosphere, 25 °C	H_2_ (6.3 mmol g^−1^), C_2_H_4_ (0.017 mmol g^−1^), C_2_H_6_ (0.25 mmol g^−1^), C_3_H_6_ (0.007 mmol g^−1^), C_3_H_8_ (0.14 mmol g^−1^), CO_2_ (5.9 mmol g^−1^), 96 h reaction	34 (2021)
1 wt% Pt loaded P25 TiO_2_	30 min plasma treatment at room temperature and atmospheric pressure with a 100 ml min^−1^ air flow	Grafting oxygen-containing functionalities on the PE/PP/PVC surface to achieve better hydrophilicity and stronger interaction between PE/PP/PVC and the catalyst	Simulated solar light (AM 1.5G, 100 mW cm^−2^), 30 mg catalyst, 30 mg PE powder, 30 ml water, N_2_ atmosphere, 25 °C	H_2_ (1056.10 μmol g^−1^), CH_4_ (93.56 μmol g^−1^), C_2_H_4_ (2.25 μmol g^−1^), C_2_H_6_ (4.25 μmol g^−1^), CO (10.99 μmol g^−1^), CO_2_ (452.43 μmol g^−1^), 24 h reaction	35 (2023)
			Simulated solar light (AM 1.5G, 100 mW cm^−2^), 30 mg catalyst, 30 mg PP powder, 30 ml water, N_2_ atmosphere, 25 °C	H_2_ (225.27 ± 6.06 μmol g^−1^), 4 h reaction	
			Simulated solar light (AM 1.5G, 100 mW cm^−2^), 30 mg catalyst, 30 mg PVC powder, 30 ml water, N_2_ atmosphere, 25 °C	H_2_ (278.56 ± 11.84 μmol g^−1^), 4 h reaction	
Nb_2_O_5_ atomic layers	—	—	300 W Xe lamp (AM 1.5G, 100 mW cm^−2^), 50 mg catalyst, 150 mg PE, 50 ml water, in air, 25 °C	Acetic acid (47.4 μg g^−1^ h^−1^), CO (0.4 μg g^−1^ h^−1^), 35 h reaction	36 (2020)
	—	—	300 W Xe lamp (AM 1.5G, 100 mW cm^−2^), 50 mg catalyst, 150 mg PP, 50 ml water, in air, 25 °C	Acetic acid (40.6 μg g^−1^ h^−1^), CO (0.3 μg g^−1^ h^−1^), 35 h reaction	
	—	—	300 W Xe lamp (AM 1.5G, 100 mW cm^−2^), 50 mg catalyst, 300 mg PVC, 50 ml water, in air, 25 °C	Acetic acid (39.5 μg g^−1^ h^−1^), CO (0.5 μg g^−1^ h^−1^), 35 h reaction	
Co-Ga_2_O_3_	—	—	300 W Xe lamp (AM 1.5G, 100 mW cm^−2^), 50 mg catalyst, 100 mg PE, 100 ml H_2_O, in air, 25 °C	H_2_ (647.8 μmol g^−1^ h^−1^), CO (158.3 μmol g^−1^ h^−1^), CO_2_ (419.3 μmol g^−1^ h^−1^), 24 h reaction	37 (2022)
	—	—	300 W Xe lamp (AM 1.5 G, 100 mW cm^−2^), 50 mg catalyst, 100 mg PP, 100 ml H_2_O, in air, 25 °C	H_2_ (603.5 μmol g^−1^ h^−1^), CO (147.2 μmol g^−1^ h^−1^), CO_2_ (389.1 μmol g^−1^ h^−1^), 24 h reaction	
	—	—	300 W Xe lamp (AM 1.5 G, 100 mW cm^−2^), 50 mg catalyst, 100 mg PET, 100 ml H_2_O, in air, 25 °C	H_2_ (384.2 μmol g^−1^ h^−1^), CO (100.6 μmol g^−1^ h^−1^), CO_2_ (258.9 μmol g^−1^ h^−1^), 24 h reaction	

**Fig. 3:**
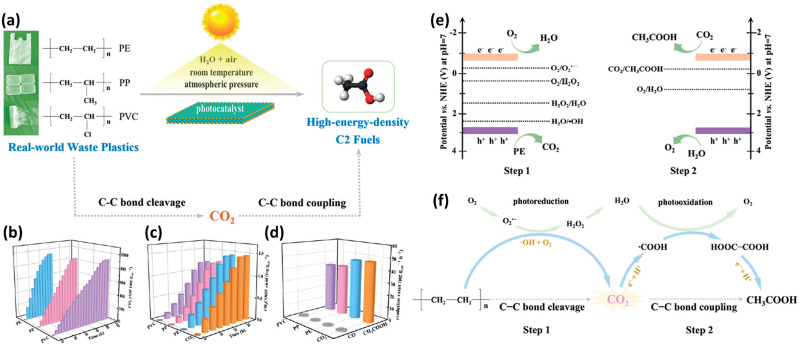
(a) Schematic figure showing the conversion of various plastic wastes into C_2_ fuels *via* a designed two-step reaction pathway under simulated natural environmental conditions. (b) Generation of CO_2_ in photocatalytic oxidation of pure PE, PP and PVC using Nb_2_O_5_ atomic layers. In this reaction, the molar ratio of carbon in each plastic and Nb_2_O_5_ atomic layers is about 50 : 1. (c) The production amounts of CH_3_COOH and (d) generation rates of CH_3_COOH and CO in photocatalytic conversion of pure PE, PP and PVC, together with the photocatalytic reduction of pure CO_2_ in water. Schematic illustration for (e) the band edge potentials of Nb_2_O_5_ atomic layers as well as the potentials for CO_2_, H_2_O, H_2_O_2_ and O_2_ redox couples at pH = 7. (f) The increased two-step C–C bond cleavage and coupling mechanism for conversion of PE into CH_3_COOH under simulated natural environmental conditions. Reproduced with permission from copyright 2020, Wiley-VCH.^36^

The Xie group^36,37^ is a pioneer in researching on photocatalytic upcycling of polyolefins and polyethylene terephthalate (PET) without a pre-treatment step. They have used two engineering strategies, *i.e.* structure regulation and element incorporation, on two metal oxide photocatalysts, Nb_2_O_5_ (ref. 36) and Ga_2_O_3_ (ref. 37), respectively, for directly photocatalytic upcycling of polyolefins and PET in an air atmosphere without pre-treatment. In one study,^36^ the authors have designed a general strategy of converting different plastic wastes (PE, PP and PVC) into CO_2_ followed by photo-reduction to form acetic acid as a C_2_ fuel under simulated natural environmental conditions (Fig. 3a). First, they designed and synthesized Nb_2_O_5_ atomic layers using the as-synthesized niobic acid atomic layers as the precursor followed by annealing in air. The earth-abundant and robust Nb_2_O_5_ is chosen due to its suitable conduction band (CB) and valence band (VB) positions (+2.5 V *vs.* SHE for the CB and −0.9 V *vs.* SHE for the VB at pH = 7). Thus, Nb_2_O_5_ can generate highly oxidative ˙OH radicals (+2.32 V *vs.* SHE at pH = 7) to degrade plastics and photo-generated electrons to reduce CO_2_ (−0.6 V *vs.* SHE at pH = 7). Nb_2_O_5_ atomic layers can degrade PE, PP and PVC with identical numbers of carbon in 40, 60 and 90 hours, respectively. The generated CO_2_ amounts increase gradually and reach the highest values in the corresponding time (Fig. 3b). They found that the overall numbers of moles of carbon in the generated CO_2_ gas and CO_2_ dissolved in solution are almost equivalent to that in pure PE, PP or PVC. These results confirm that plastics are completely degraded to form CO_2_ gas. Furthermore, the generated CH_3_COOH amounts are also gradually increased (Fig. 3c) and averaged CH_3_COOH generation rates on PE, PP and PVC are ∼47.4, 40.6 and 39.5 μg g^−1^ h^−1^, respectively (Fig. 3d). To obtain insightful understanding of the reaction mechanism on photocatalytic conversion of plastics into CH_3_COOH, *in situ* electron spin resonance (ESR) spectra were collected. The results confirm the generation of ˙OH and ˙O_2_^−^ radicals in photocatalytic PE conversion. Furthermore, to study the accurate origin of generated CO_2_, synchrotron-radiation vacuum ultraviolet photoionization mass spectrometry (SVUV-PIMS) was conducted to study the reaction products in photocatalytic PE conversion under simulated natural conditions with a low amount of H_2_^18^O. The results reveal that H_2_O can participate in both the photo-oxidation procedures to generate CO_2_ and O_2_, respectively. To seek the origin of generated CH_3_COOH, *in situ* FTIR spectroscopy was conducted. The results exhibit the formation of ˙COOH intermediates in photocatalytic PE conversion. Then, the C–C coupling of neighbouring ˙COOH leads to the formation of HOOC–COOH intermediates. Then, the continuous protonation of HOOC–COOH occurs to form HOOC–CO˙, HOOC–CHO˙ and HOOC–CH_2_O˙ intermediates as well as CH_3_COOH finally. Based on the above results, as shown in Fig. 3e and f, they propose the mechanism for photoconversion of pure PE into CH_3_COOH as follows: (i) the O_2_ and ˙OH radical mediated oxidative C–C bond cleavage to yield CO_2_ using Nb_2_O_5_ atomic layers, whilst photo-reduction of O_2_ occurs to generate ˙O_2_^−^, H_2_O_2_ and H_2_O. (ii) Light induced C–C coupling of ˙COOH intermediates generates CH_3_COOH, whilst H_2_O is oxidized to form O_2_. In another study, the Xie group^37^ has synthesized Co doped Ga_2_O_3_ nanosheets (Co-Ga_2_O_3_) for photocatalytic conversion of pulverized powder from PE bags, PP boxes or PET bottles into syngas (CO and H_2_) along with CO_2_ in the presence of water under ambient conditions. First, they reveal the CB and VB edge positions for both Co-Ga_2_O_3_ and Ga_2_O_3_, confirming that both of their photo-induced electrons and holes can drive some pivotal reactions, *e.g.*, H_2_O oxidation or CO_2_/O_2_/H_2_O reduction. Then PE bags, PP boxes and PET plastic bottles were crushed into powders using a pulveriser. Afterwards, Co-Ga_2_O_3_ or Ga_2_O_3_ was utilized to convert these powders in pure water with simulated solar light (AM 1.5G, 100 mW cm^−2^) at ambient temperature and pressure *via* a photocatalysis reaction. ^1^H NMR spectroscopy reveals no detectable liquid product and H_2_, CO and CO_2_ were found by gas chromatography (GC). Especially, Co-Ga_2_O_3_ exhibits the photocatalytic evolution activities of H_2_ (647.8 μmol g^−1^ h^−1^) and CO (158.3 μmol g^−1^ h^−1^) from converting PE powders, about 160 and 190% times larger than those of Ga_2_O_3_. After 48 hour irradiation, the weight loss of PE bags can reach 81%. Co-Ga_2_O_3_ also exhibits excellent stabilities for photocatalytic conversion of these plastic powders. Control experiments show that the generated H_2_ arises from H_2_O rather than plastics; both O_2_ and H_2_O participated in the oxidation of PE to CO_2_, which is further reduced to form CO. The *in situ* electron spin resonance (ESR) technique confirms the existence of ˙OH and ˙O_2_^−^ radicals with Co-Ga_2_O_3_ or Ga_2_O_3_ in photocatalytic reactions. Combining with isotope-labelling experiments, they deduce that both the ˙OH radical and O_2_ are involved in the photocatalytic oxidation of PE to CO_2_. *In situ* FTIR spectra further confirm that Co-Ga_2_O_3_ can reduce CO_2_ to CO *via* a photocatalytic reaction. Based on the revealed results, they propose the photocatalytic mechanism as below: (i) with light illumination, the photo-induced electrons/holes in Co-Ga_2_O_3_ or Ga_2_O_3_ can react with water to form H_2_ and O_2_, respectively; (ii) ˙OH radicals and O_2_ are involved in converting plastics into CO_2_, whilst O_2_ was also reduced to form ˙O_2_^−^ radicals, H_2_O_2_ and H_2_O; (iii) the formed CO_2_ is further reduced to generate CO and O_2_ is also formed *via* oxidation of H_2_O. Furthermore, the reasons for the increased activities of Co-Ga_2_O_3_ compared to those of Ga_2_O_3_ are summarized as follows: (i) the enhanced light absorption of Co-Ga_2_O_3_ from d–d internal transition; (ii) the increased density of states (DOS) at the VB edge for Co-Ga_2_O_3_; (iii) the obviously reduced charge recombination for Co-Ga_2_O_3_; (iv) the stronger adsorption towards CO_2_ for Co-Ga_2_O_3_; (v) the lower energy barrier for CO_2_ reduction or H_2_ evolution on Co-Ga_2_O_3_. Both of the above two studies have adopted a one-step photocatalytic upcycling route to directly convert polyolefins into value-added chemicals in an air atmosphere. With the existence of O_2_ in air, various ROSs can be generated to participate in the reactions, which can help convert these plastics into oxygenated value-added chemicals/fuels and CO_2_. The generated CO_2_ can be further reduced by photo-induced electrons to form value-added chemicals, *e.g.*, CO and acetic acid. All the performances and reaction conditions of the studies in this section are summarized in Table 1.

### 3.2 Metal sulphide based photocatalysts for plastic upcycling

Up till now, most metal sulphide based photocatalysts reported for plastic upcycling are based on Cd-based catalysts,^38–40^ such as CdS QD^38^ and Cd_*x*_Zn_1−*x*_S solid solution,^40^ because of their strong light absorption arising from the appropriate band gap, together with their excellent reduction abilities due to the favourable CB edge positions.^38–40^ Thus, they usually exhibit outstanding H_2_ evolution rates under visible light irradiation under anaerobic conditions when applied on photocatalytic plastic upcycling. On the other hand, these metal sulphides possess moderate oxidation abilities owing to the S 3p derived VB. Thus, their photo-induced holes can oxidize the pre-treated plastics without over-oxidation to form CO_2_ under anaerobic conditions. However, owing to their moderate oxidation abilities, it is challenging for metal sulphide based photocatalysts to directly upcycle the plastics without pre-treatment. The following studies in this section all focus on photocatalytic upcycling of polyesters (*e.g.*, PET, PLA and PUR) and polyolefins (*e.g.*, PE) after pre-treatment, in which these polymers with large molecular weights are first transformed into monomers/oligomers or short-chain carbon-based molecules.^38–40^ Several strategies, such as surface engineering,^38^ loading cocatalysts^39^ and band structure engineering,^40^ have been adopted to increase the photocatalytic efficiencies.

**Fig. 4:**
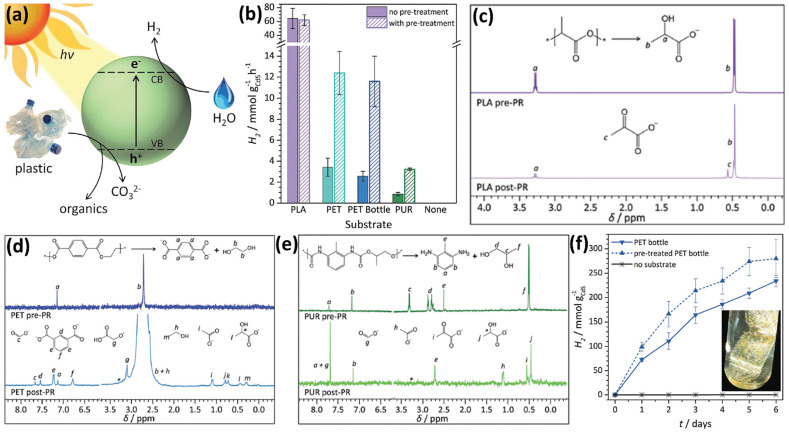
(a) Schematic illustration for photo-reforming various plastic wastes using CdS/CdO_*x*_ QDs in alkaline aqueous solution. (b) Photocatalytic reforming of various plastics by CdS/CdO_*x*_ QDs. Reaction conditions: 1 nmol CdS/QDs, plastic powders (50 mg ml^−1^ PLA, 25 mg ml^−1^ PET, PET bottle or PUR), without pre-treatment or pre-treated in 2 ml 10 M NaOH aqueous solution. ^1^H-NMR spectra for (c) PLA, (d) PET and (e) PUR prior to (pre-PR) and after (post-PR) 24 hour light illumination using 1 nmol CdS/CdO_*x*_ QDs in 2 ml 10 M NaOD in D_2_O. (f) Photocatalytic reforming of the PET bottle to generate H_2_ using CdS/CdO_*x*_ QDs. Reaction conditions: 1 nmol CdS/CdO_*x*_ QDs, ground PET bottle (25 mg ml^−1^) directly used or pre-treated in 2 ml 10 M NaOH aqueous solution. (f) Inset shows the image of the PET bottle and H_2_ bubbles on the surface of plastics. Reproduced with permission from copyright 2018, Royal Society of Chemistry.^38^

The Reisner^38^ group has presented a strategy to photo-reform plastic wastes to yield H_2_ and value-added organics using photocatalysts in water under sunlight (Fig. 4a). They have designed and synthesized a CdS/CdO_*x*_ quantum dot (QD) photocatalyst. They found that when CdS QDs were added into aqueous NaOH, a thin cadmium oxide/hydroxide (CdO_*x*_) is generated to impede photo-corrosion. Ligand-free QDs are found to work with most substrates due to their exposed surfaces. In comparison, oleic acid-capped QDs can only work with PET, probably owing to the hydrophobic effect benefiting the substrate–QD interaction. First, CdS/CdO_*x*_ was adopted for photocatalytic reforming of various polymers including polylactic acid (PLA), PET, polyurethane (PUR), polyvinylpyrrolidone (PVP), polyethylene glycol (PEG), LDPE, PVC, poly(methyl methacrylate) (PMMA), polystyrene (PS) and polycarbonate (PC), respectively. Among them, only PLA, PET and PUR are found to achieve higher photocatalytic H_2_ evolution performances while much less photocatalytic H_2_ evolution is observed on the other substrates. So these three polymers are selected for photocatalytic reforming. CdS/CdO_*x*_ QDs were used for photocatalytic reforming of PLA, PET and PUR in NaOH aqueous solution in a N_2_ atmosphere. With optimised reaction conditions, CdS/CdO_*x*_ QDs exhibit the photocatalytic H_2_ evolution activities of 64.3 ± 14.7, 3.42 ± 0.87 and 0.85 ± 0.28 mmol g^−1^ h^−1^, respectively. Isotope-labelling experiments reveal that the H_2_ produced arises from water, not the substrate. As a comparison, 5% Pt loaded TiO_2_ only exhibits the H_2_ evolution rates of 0.011 ± 0.004 and 0.074 ± 0.029 mmol g^−1^ h^−1^ under identical reaction conditions. And without expensive Pt as the co-catalyst, bare TiO_2_ exhibits no H_2_ evolution. Besides, ZnSe QDs as a Cd-free catalyst show no H_2_ production under the same reaction conditions. These highlight the advantages of strong visible-light absorption, no use of co-catalysts and fast oxidation of complicated substrates for CdS/CdO_*x*_ QDs. To further increase the rate, a pre-treatment route was developed to hydrolyse PET, PUR and PLA in 10 M NaOH aqueous solution for 24 hours at 40 °C to release the monomers. After pre-treatment and removing the undissolved polymer by centrifugation, the supernatant was used to reduce the absorbance and scattering, thus leading to more photons absorbed by CdS/CdO_*x*_ QDs and higher rates. Pre-treated PET and PUR solutions were found to obviously increase the activities of CdS/CdO_*x*_ QDs, compared to Raw PET and PUR, respectively (Fig. 4b). Compared with Raw PLA, pre-treated PLA exhibits almost no influence on the efficiency of CdS/CdO_*x*_ QDs (Fig. 4b). This is because PLA is easily dissolved in NaOH aqueous solution. Another advantage of CdS/CdO_*x*_ QDs is that they can function even in highly alkaline solution. Then, ^1^H-NMR spectroscopy is adopted to study the reaction solutions and organic oxidation products. It was found that PLA is first hydrolysed to form sodium lactate followed by oxidation to generate a pyruvate-based compound (Fig. 4c). As for PET, it is first hydrolysed to generate terephthalate, ethylene glycol and isophthalate, followed by the formation of formate, glycolate, ethanol, acetate and lactate (Fig. 4d). Photo-reforming terephthalic acid doesn't generate H_2_, indicating that only the aliphatic component of PET yields the oxidation products. And terephthalic precipitates as a disodium salt to be easily recovered as a valuable chemical. As shown in Fig. 4e, PUR is hydrolysed to form an aliphatic component (propylene glycol) and aromatic component (2,6-diaminotoluene). While propylene glycol is oxidized to form formate, acetate, pyruvate and lactate (Fig. 4e), 2,6-diaminotoleune remains intact. The overall conversion of all polymers is lower than 40%, since CdS/CdO_*x*_ QDs cannot completely mineralise these polymers into CO_2_. And no CO_3_^2−^ or CO_2_ is detected. These polymers are just partially oxidized to form various chemicals. CdS/CdO_*x*_ QDs are also shown to achieve photo-reforming of a PET bottle and pre-treated PET bottle to generate H_2_ and value-added chemicals (Fig. 4f).

**Fig. 5:**
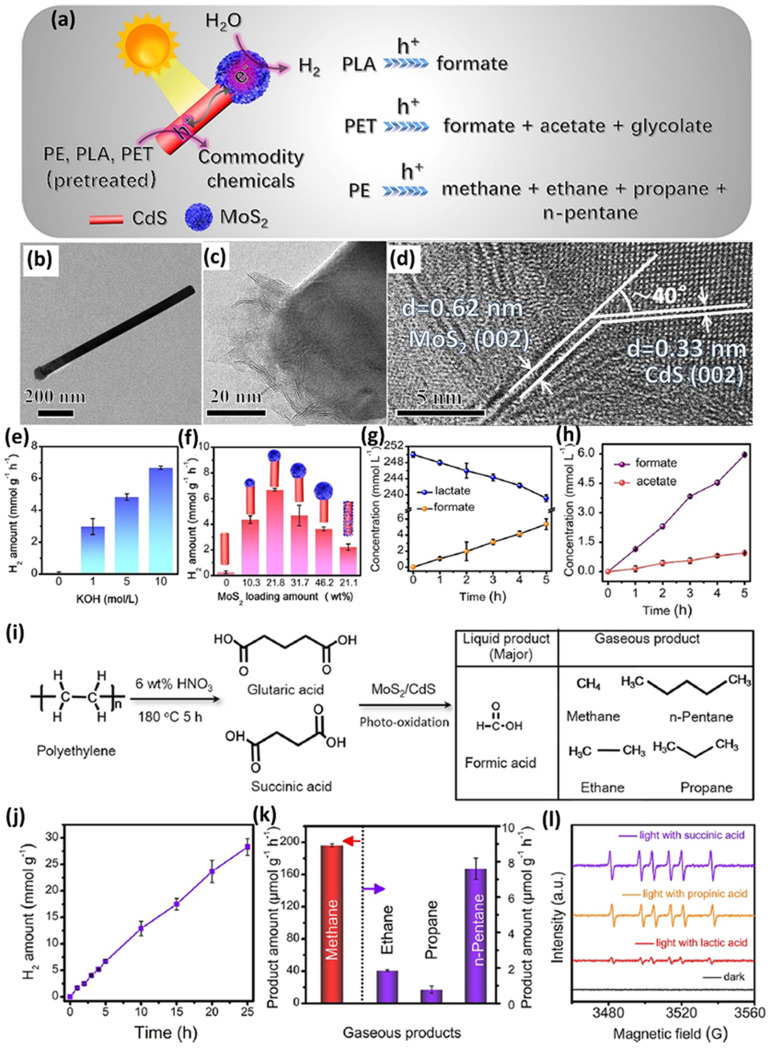
(a) Schematic illustration for photocatalytic reforming of pre-treated plastics using the MoS_2_/CdS composite. (b and c) TEM images and (d) HRTEM image of the MoS_2_/CdS composite. (e) Photocatalytic reforming of pre-treated PLA for H_2_ evolution using 21.8 wt% MoS_2_ loaded CdS in various KOH concentrations. (f) Effect of MoS_2_ loading on the H_2_ evolution from photocatalytic reforming of pre-treated PLA in 10 M KOH using MoS_2_/CdS composites with different MoS_2_ contents. (g) The concentrations of lactate and formate in a 5 hour photo-reforming reaction. (h) The concentrations of acetate and formate in a 5 hour photo-reforming reaction. (i) Schematic image showing the conversion of PE into carboxylic acid followed by a photo-reforming reaction on MoS_2_/CdS. (j) H_2_ generation from 25 hour photocatalytic reforming of pre-treated PE. (k) Generation activities of alkane from photocatalytic reforming of pre-treated PE in a 5 hour reaction. (l) *In situ* ESR spectra in the presence of various substrates *via* using the spin-trapping agent. Reproduced with permission from copyright 2022, American Chemical Society.^39^

In another study, a cocatalyst/photocatalyst MoS_2_/CdS system was synthesized for photocatalytic upcycling of pre-treated polyester/polyolefin.^39^ The Qiu group^39^ designed this system, MoS_2_-tipped CdS nanorod (MoS_2_/CdS), to reform the pre-treated PE, PLA or PET to generate various value-added chemicals and H_2_, as shown in Fig. 5a. The as-synthesized MoS_2_/CdS composite exhibits the accumulation of photo-generated electrons and holes on the MoS_2_ tip and sidewalls of CdS, respectively (Fig. 5a). The TEM image (Fig. 5b and c) and HRTEM image (Fig. 5d) directly reveal the intimate coupling of MoS_2_ on the tip of CdS NSs. The strong electronic coupling between MoS_2_ and CdS is confirmed by the surface-sensitive high-resolution XPS technique, showing that electrons are transferred from CdS to MoS_2_. Results of steady-state PL, photocurrent response and electrochemical impedance spectroscopy (EIS) all confirm the more efficient charge separation/migration in MoS_2_/CdS. Furthermore, selective deposition reactions indicate that MnO_*x*_ nanosheets and Pt nanoparticles are selectively loaded on the sidewalls of CdS and the MoS_2_ tip, respectively. These results also indicate that photo-generated electrons and holes are accumulated on the MoS_2_ tip and sidewalls of CdS, respectively. Linear sweep voltammetry (LSV) curves further indicate the significantly higher HER activity of MoS_2_/CdS compared to MoS_2_ alone. Then, the electron spin resonance (ESR) technique is further utilized to detect the active species on MoS_2_/CdS, which confirms the existence of photo-generated holes and ˙OH with light illumination. Nevertheless, a photoluminescence test using terephthalic acid (TPA) as the ˙OH scavenger only shows a negligible characteristic PL peak arising from ˙OH, suggesting the minimal role of ˙OH in photo-reforming. In contrast, photo-generated holes are deemed as the major active species in photo-reforming. The MoS_2_/CdS photocatalyst was utilized for photo-reforming of pre-treated PLA, PET and PE. The largest H_2_ evolution of 6.68 ± 0.10 mmol g^−1^ h^−1^ was realized by MoS_2_/CdS from photo-reforming of pre-treated PLA in 10 M NaOH aqueous solution (Fig. 5e). Fig. 5f shows the influence of various MoS_2_ loading amounts on the tip of CdS, with 21.8 wt% MoS_2_ loading reaching the highest H_2_ evolution activity. To highlight the unique benefit of the MoS_2_/CdS structure, MoS_2_ nanosheets selectively loaded on the sidewalls of CdS nanorods were synthesized as the control sample, denoted as CdS@MoS_2_. In contrast, CdS@MoS_2_ with a similar loading of 21.1 wt% only exhibits a much lower photocatalytic H_2_ evolution rate of 2.21 ± 0.25 mmol g^−1^ h^−1^, compared with that of MoS_2_/CdS under identical reaction conditions (Fig. 5f). MoS_2_/CdS exhibits 200 hour long-term stability for photocatalytic H_2_ evolution in pre-treated PLA solution, with 72% of the activity in the 8th cycle compared with that in the 1st cycle. Furthermore, isotope labelling experiments confirm that the source of generated H_2_ is water splitting, but not the pre-treated PLA substrate. Both ^1^H NMR spectroscopy and high-performance liquid chromatography (HPLC) together indicate the generation of formate (5.37 ± 0.67 mmol l^−1^) after 5 hour light illumination (Fig. 5g). ^13^C NMR spectroscopy and the self-built route together confirm the production of CO_3_^2−^ arising from lactate oxidation by MoS_2_/CdS. Additionally, DMPO and Na_2_S/Na_2_SO_3_ are added as scavengers of ˙OH radicals and Na_2_S/Na_2_SO_3_, respectively. No obvious reduction of formate was observed after adding DMPO. In contrast, apparent reduction of formate was found after adding Na_2_S/Na_2_SO_3_, indicating the principal role of holes in lactate oxidation. This is in accordance with the ESR and PL results. Then, it is proposed that lactate is oxidized by holes to form acetaldehyde followed by acetate, methanol and formate. But the other products are not observed by ^1^H NMR spectroscopy and HPLC except formate. They have also tested the photocatalytic reforming of PET on MoS_2_/CdS, displaying stable H_2_ evolution in a 25 hour test, with a rate of up to 3.90 ± 0.07 mmol g^−1^ h^−1^. The 200 hour test further indicates the structural and composition stability of MoS_2_/CdS. Isotope-labelling experiments suggest that the generated H_2_ arises from water splitting, but not from PET constituent monomers. Furthermore, photocatalytic reforming of a pre-treated PET bottle shows a H_2_ evolution rate of 3.53 ± 0.07 mmol g^−1^ h^−1^, further confirming the potential realistic application. Formate, acetate and glycolate can also be detected by ^1^H NMR spectroscopy in photo-reforming of pre-treated PET. The gradual increase of formate and acetate amounts can be observed in a 5 hour reaction (Fig. 5h). The ^1^H NMR test shows that the concentration of terephthalate is not changed but the concentration of ethylene glycol is altered, suggesting that these formed carboxylate chemicals arise from ethylene glycol but not from terephthalate. Holes were confirmed to be the major active species for ethylene glycol oxidation *via* control experiments. MoS_2_/CdS was also utilized for photo-reforming of the pre-treated PE by nitric acid in a hydrothermal reaction (Fig. 5i). HPLC tests confirm the existence of formic acid, succinic acid, glutaric acid, acetic acid, propionic acid and adipic acid, with the major products of succinic acid and glutaric acid (Fig. 5i). Then, MoS_2_/CdS was utilized to photo-reform the pre-treated PE, generating a H_2_ evolution rate of 1.13 ± 0.06 mmol g^−1^ h^−1^ (Fig. 5j). Notably, MoS_2_/CdS still exhibits robust H_2_ evolution after a 200 hour test, with good PET compositional/structural stability for the reacted MoS_2_/CdS. Isotope-labelling experiments also reveal that most of the generated H_2_ arises from water splitting. Besides, MoS_2_/CdS also shows a CH_4_ generation rate of up to 196.2 ± 1.76 μmol g^−1^ h^−1^ (Fig. 5k) and a CO_2_ generation rate of 2.75 ± 0.05 μmol g^−1^ h^−1^. Control experiments reveal that CH_4_ originates from the Kolbe photo-oxidation decarboxylation of carboxylic acid, not from CO_2_ reduction. Due to the existence of abundant carboxylic acids in substrates, other gaseous alkanes, such as ethane, propane and *n*-pentane, are also generated with rates of 1.86 ± 0.04, 0.78 ± 0.20 and 7.6 ± 0.60 μmol g^−1^ h^−1^ (Fig. 5k), respectively, *via* decarboxylation with a hydrogen transfer mechanism. An *in situ* ESR test confirms the production of carbon-centred radical species, deemed as the pivotal intermediates in the Kolbe decarboxylation reaction (Fig. 5l). These results confirm that MoS_2_/CdS can induce the decarboxylation reaction. Control experiments further indicate that holes play a key role in photocatalytic decarboxylation. Finally, they propose a mechanism: (i) photo-induced holes lead to the oxidation of acetic acid to generate methane *via* decarboxylation; (ii) photocatalytic decarboxylation of succinic acid and glutaric acid leads to generation of ethane and propane, respectively. This successful photocatalytic decarboxylation reaction is for the first time reported on CdS based photocatalysts. Similarly, Li *et al.*^40^ combined the strategies of band structure engineering and loading cocatalysts to synthesize 4.3 wt% MoS_2_ coupled Cd_0.5_Zn_0.5_S (M_4.3_/C_0.5_Z_0.5_S). The strong electronic coupling between MoS_2_ and Cd_0.5_Zn_0.5_S was confirmed by the obvious peak shifts in Raman and XPS spectra. Transient surface photovoltage (TR-SPV) results show the 250% higher SPV value of M_4.3_/C_0.5_Z_0.5_S compared to that of Cd_0.5_Zn_0.5_S, as well as the elongated SPV signal of M_4.3_/C_0.5_Z_0.5_S. These results indicate the much more efficient charge separation and longer lifetimes of photo-induced charge carriers in M_4.3_/C_0.5_Z_0.5_S. These are further corroborated by M_4.3_/C_0.5_Z_0.5_S *via* the electrochemical impedance spectra (EIS) and photoelectrochemical current (PEC) densities. The polarization curves of M_4.3_/C_0.5_Z_0.5_S exhibit increased HER activity compared to that of Cd_0.5_Zn_0.5_S, indicating that the loading of MoS_2_ can increase the activities of M_4.3_/C_0.5_Z_0.5_S. Blank experiments show that without light, a photocatalyst, or NaOH, H_2_ evolution or degradation of PET cannot occur. M_4.3_/C_0.5_Z_0.5_S shows the largest H_2_ generation activity (15.9 mmol h^−1^ g^−1^) compared to 4.3 wt% MoS_2_ coupled Cd_*x*_Zn_1−*x*_S (*X* = 0, 0.2, 0.4, 0.8 and 1). An outstanding photocatalytic H_2_ generation rate was also realized by M_4.3_/C_0.5_Z_0.5_S using PET bottles. Good H_2_ evolution robustness was also observed in 5 hour irradiation in PET or PET-bottle-based aqueous solution. ^1^H-NMR spectroscopy was adopted to analyse the degradation products of photocatalytic PET conversion. Ethylene glycol, terephthalic acid and glycolate were detected in the pre-treated solutions before reaction. Finally, the pre-treated PET was oxidized to yield formate, methanol, acetate and ethanol.

The above three studies have demonstrated the immense potential of metal sulphide based photocatalysts, especially Cd-based catalysts, to upcycle these pre-treated plastics into valuable chemicals/fuels using simulated solar light. But the toxicity of Cd-based catalysts, together with the insufficient stability and oxidation capacity of metal sulphide based photocatalysts, seriously restricts their realistic applications in industrial scale solar plastic upcycling. All the performances and reaction conditions of the studies in this section are summarized in Table 2.

**Table 2:** Metal sulphide based photocatalysts for photo-reforming of plastics

Metal sulphide based catalyst	Pre-treatment	Product after pre-treatment	Reaction conditions	Activity and stability	Reference (year)
CdS/CdO_*x*_ quantum dots	50 mg ml^−1^ PLA, 2 ml 10 M NaOH aqueous solution, 40 °C, 24 h, in air	PLA converted into sodium lactate	Simulated solar light (AM 1.5G, 100 mW cm^−2^), 2 ml pre-treated solution, 25 °C, N_2_ atmosphere	H_2_ (62.1 ± 7.8 mmol g^−1^ h^−1^), pyruvate generated, 4 h reaction	38 (2018)
	25 mg ml^−1^ PET, 2 ml 10 M NaOH aqueous solution, 40 °C, 24 h, in air	PET converted into terephthalate, ethylene glycol and isophthalate		H_2_ (12.4 ± 2.0 mmol g^−1^ h^−1^) formate, glycolate, ethanol, acetate and lactate generated, 4 h reaction	
	25 mg ml^−1^ PUR, 2 ml 10 M NaOH aqueous solution, 40 °C, 24 h, in air	PUR converted into 2,6-diaminotoluene and propylene glycol		H_2_ (3.22 ± 0.13 mmol g^−1^ h^−1^) formate, acetate, pyruvate and lactate generated, 4 h reaction	
MoS_2_-tipped CdS nanorod	1.5 g PLA, 60 ml 10 M KOH solution, 40 °C, 48 h	PLA converted to lactate	300 W Xe light, simulated solar light (AM 1.5G, 100 mW cm^−2^), 100 mg catalyst, 5 °C, anaerobic conditions (vacuumed)	H_2_ (6.20 ± 0.23 mmol g^−1^ h^−1^), 25 h reaction, H_2_ (6.20 ± 0.23 mmol g^−1^ h^−1^), 200 h reaction, formate (5.37 ± 0.67 mmol l^−1^), 5 h reaction	39 (2022)
	1.5 g PET, 60 ml 10 M KOH solution, 40 °C, 4 h	PET converted to ethylene glycol and terephthalate		H_2_ (3.90 ± 0.07 mmol g^−1^ h^−1^), 25 h reaction, formate (5.96 ± 0.02 mmol l^−1^), acetate (0.95 ± 0.01 mmol l^−1^), 5 h reaction	
	1.5 g PE, 60 ml 6 wt% HNO_3_, hydrothermal reaction at 180 °C for 5 h	PE converted to formic acid, succinic acid, glutaric acid, acetic acid, propionic acid, adipic acid		H_2_ (1.13 ± 0.06 mmol g^−1^ h^−1^), 25 h reaction, CH_4_ (196.2 ± 1.76 μmol g^−1^ h^−1^), C_2_H_6_ (1.86 ± 0.04 μmol g^−1^ h^−1^), C_3_H_8_ (0.78 ± 0.2 μmol g^−1^ h^−1^), *n*-pentane (7.60 ± 0.6 μmol g^−1^ h^−1^) CO_2_ (2.75 ± 0.05 μmol g^−1^ h^−1^), 5 h reaction	
MoS_2_–Cd_0.5_Zn_0.5_S	1.5 g PET, 60 ml 10 M NaOH aqueous solution, 40 °C, 24 h	PET converted to ethylene glycol, terephthalic acid, and glycolate	300 W Xe light (AM 1.5G), 10 mg catalyst, 60 ml pre-treated PET solution, anaerobic conditions (vacuumed)	H_2_ (15.90 mmol g^−1^ h^−1^) formate, methanol, ethanol and acetate generated	40 (2021)

### 3.3 Non-metal based photocatalysts for plastic upcycling

**Fig. 6:**
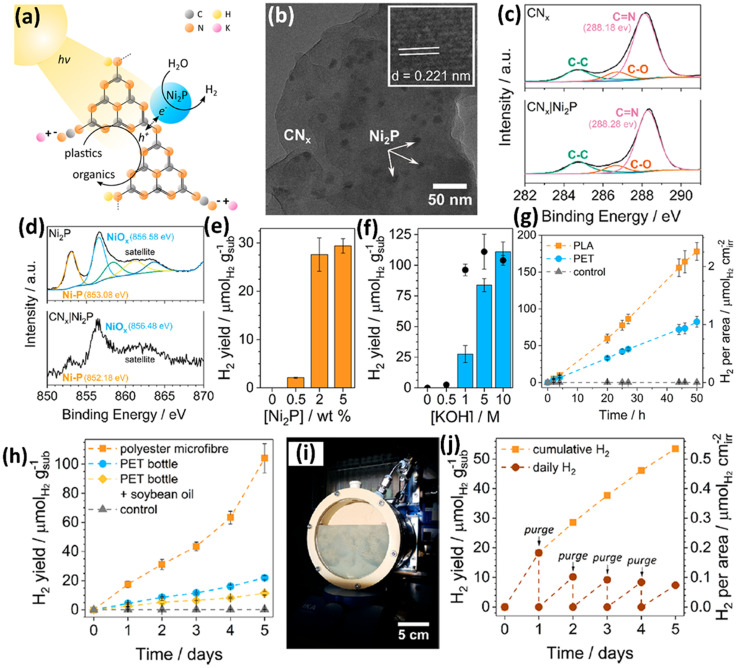
(a) Schematic illustration of plastic photo-reforming using a Ni_2_P/CN_*x*_ photocatalyst. (b) TEM image of Ni_2_P/CN_*x*_. (b) Inset shows the lattice distance of Ni_2_P NPs. (c) High resolution XPS spectra of C 1s for CN_*x*_ and Ni_2_P/CN_*x*_. (d) High resolution XPS spectra of Ni 2p for Ni_2_P and Ni_2_P/CN_*x*_. (e) Effect of Ni_2_P loading on the H_2_ evolution activities of Ni_2_P/CN_*x*_ for 20 hour PET photo-reforming. (f) Effect of KOH concentration on the H_2_ evolution activities of Ni_2_P/CN_*x*_ for 20 hour PET photo-reforming. (g) Long-term photocatalytic reforming of PET and PLA. Reaction conditions: 2 wt% Ni_2_P/CN_*x*_ (1.6 mg ml^−1^), pre-treated PET (25 mg ml^−1^), 2 ml 1 M KOH aqueous solution, simulated sunlight (AM 1.5G, 100 mW cm^−2^) and 25 °C. (h) Long term photocatalytic reforming of polyester microfibers, a PET bottle and a PET bottle coated with soybean oil. Reaction conditions: 1.6 mg ml^−1^ Ni_2_P/CN_*x*_, 2 ml 1 M KOH, 5 mg ml^−1^ pre-treated microfibers, 25 mg ml^−1^ pre-treated PET bottle or 25 mg ml^−1^ pre-treated PET bottle with 5 mg ml^−1^ soybean oil and simulated sunlight (AM 1.5G, 100 mW cm^−2^). (i) Image of the upscaled photocatalytic reactor. (j) Upscaled photocatalytic reforming of polyester microfibers. Reaction conditions: 1.6 mg ml^−1^ Ni_2_P/CN_*x*_, 120 ml 1 M KOH, 5 mg ml^−1^ pre-treated microfibers and simulated sunlight (AM 1.5G, 100 mW cm^−2^). Reproduced with permission from copyright 2019, American Chemical Society.^41^

Currently, all the non-metal based photocatalysts are based on carbon nitride (C_*x*_N_*y*_), which possess non/low toxicity, earth abundance, low cost, good light absorption due to a suitable band gap width, favourable redox abilities arising from appropriate CB/VB edge positions and strong chemical-/photo-stability.^41–44^ Owing to the moderate oxidation ability of C_*x*_N_*y*_, it is very challenging for them to directly upcycle plastics into value-added chemicals/fuels at room temperature and under anaerobic conditions. Thus, the Reisner group have developed a cocatalyst/photocatalyst (Ni_2_P loaded CN_*x*_) system to photo-reform various pre-treated plastics to acquire value-added chemicals without generating CO_2_ or even CO_3_^2−^.^41^ They have reported the photo-reforming of plastic wastes to produce value-added organics and H_2_ using a Ni_2_P loaded cyanamide-functionalized carbon nitride (^H2N^CN_*x*_|Ni_2_P) photocatalyst (Fig. 6a). The TEM image of ^H2N^CN_*x*_|Ni_2_P (Fig. 6b) shows the loading of Ni_2_P NPs on the surface of ^H2N^CN_*x*_. A lattice distance of 0.221 nm was observed in the Fig. 6b inset, attributed to the (111) facet of hexagonal N_2_P. No obvious change is detected between the high resolution XPS spectra of C 1s for ^H2N^CN_*x*_ and ^H2N^CN_*x*_|Ni_2_P, suggesting that loading the Ni_2_P co-catalyst doesn't impose obvious influence on the surface features of ^H2N^CN_*x*_. The high-resolution XPS spectra of Ni 2p_3/2_ for ^H2N^CN_*x*_|Ni_2_P obviously shift to the lower binding energy direction, compared to those for Ni_2_P, suggesting a strong cocatalyst–support interaction with electrons transferring from ^H2N^CN_*x*_ to Ni_2_P. First, they pre-treated PET and PLA in KOH aqueous solution at 40 °C for 24 hours to acquire the corresponding monomers (ethylene glycol and terephthalate for PET; lactate for PLA). Quantitative analysis using ^1^H nuclear magnetic resonance (NMR) spectroscopy indicates that 72% of lactate in PLA as well as 62% of ethylene glycol and 51% of terephthalate in PET are released in pre-treatment. Then, all the reaction conditions, such as the loading of the Ni_2_P co-catalyst (Fig. 6e) and concentration of KOH (Fig. 6f), were optimized to achieve the highest photocatalytic H_2_ evolution. 50 hour solar light illumination on ^H2N^CN_*x*_|Ni_2_P leads to photocatalytic H_2_ evolution of 82.5 ± 7.3 and 178 ± 12 μmol g^−1^ using pre-treated PET and PLA as the substrates, respectively, in 1 M KOH aqueous solution. CN_*x*_|Ni_2_P without cyanamide functionalization exhibits lower H_2_ evolution using pre-treated PET or PLA as the substrate under the same conditions. Nevertheless, ^H2N^CN_*x*_|Ni_2_P shows reduced H_2_ evolution over time; CN_*x*_|Ni_2_P exhibits more stable H_2_ evolution. ^1^H NMR spectroscopy combined with ^13^C NMR spectroscopy indicates that CN_*x*_|Ni_2_P can photo-reform the pre-treated PET to generate formate, glyoxal, glycolate, acetate, glyoxylate and glycolaldehyde. Besides, CN_*x*_|Ni_2_P can photo-reform the pre-treated PLA into formate and acetate. CN_*x*_|Ni_2_P also exhibits the feasibility of photo-reforming real-world waste of polyester microfiber, a PET bottle and an oil-contaminated PET bottle into H_2_ and a series of chemicals (Fig. 6h). Post-catalysis characterization indicates the good stability of CN_*x*_ in CN_*x*_|Ni_2_P. However, it should be noted that while 99.1% Ni content of Ni_2_P still remains on CN_*x*_, XPS results show that at least the surface of Ni_2_P is converted to Ni(OH)_2_ after reaction in the KOH aqueous solution. Then, they further upscaled the photocatalytic reactor to 120 ml (Fig. 6i). Using this upscaled reactor, a photocatalytic H_2_ evolution of 53.5 μmol g_sub_^−1^ was achieved *via* photo-reforming of polyester microfibers in five days (Fig. 6j). Furthermore, the Reisner group^42^ has assembled a photocatalyst panel loaded with CN_*x*_|Ni_2_P to photo-reform various wastes including plastics. They utilized a simple low temperature and drop casting route to synthesize scalable photocatalyst panels with carbon nitride/nickel phosphide (CN_*x*_/Ni_2_P). Ni_2_P nanoparticles were dispersed on CN_*x*_ as the co-catalyst. First, they optimised the CN_*x*_/Ni_2_P panels for the highest H_2_ evolution, light harvesting and recyclability on a scale of 1 cm^2^. Afterwards, they adopted a 25 cm^2^ panel to photo-reform PET, α-cellulose and municipal solid waste (MSW), respectively. They found that the illumination configuration (front illumination or back illumination) plays the key role in the H_2_ evolution activities. To demonstrate the feasibility of “real world” application of this system, it is operated in seawater and 20% sunlight irradiation (20 mW cm^−2^), and still shows ∼50% H_2_ evolution activity compared to that in pure water and 1 sunlight irradiation (100 mW cm^−2^).

The Yang group^43^ has fabricated a graphitic carbon nitride (CN)/carbon nanotubes (CNTs)/NiMo nanoparticle (CN/CNTs/NM) photocatalyst for photo-reforming of pre-treated PET. The intimate combination between CN and *in situ* generated CNTs was confirmed by the FTIR, Raman and TGA techniques. CN/CNTs/NM shows the highest activity for H_2_ evolution from photo-reforming of PET. Carbon nitride (CN), carbon nitride/carbon nanotubes (CN/CNTs) and CN/CNTs/NM were utilized to photo-reform pre-treated PET or PLA. The ^1^H NMR spectrum confirms the existence of ethylene glycol (EG), terephthalate (TPA) and other small molecules after the pre-treatment of PET. The ^1^H NMR technique also confirms the formation of glyoxal and glycolate after photo-reforming of pre-treated PET. The highest H_2_ evolution was observed on CN/CNTs/NM for photo-reforming of PET, about 14 times larger than that of CN. Additionally, CN/CNTs/NM exhibits higher H_2_ evolution activity for photo-reforming of PLA compared to that for photo-reforming of PET. CN/CNTs/NM also exhibits good robustness for H_2_ evolution from photo-reforming of pre-treated PET. CN/CNTs/NM was also adopted to photo-reform a pre-treated PET bottle, which shows slightly smaller activity compared to that of pure PET, due to the existence of many different additives in the PET bottle. Single-particle PL tests further confirm that the PL of CN/CNTs/NM is obviously quenched compared to that of CN/CNTs or CN. The PL of CN/CNTs is also quenched compared to CN. These results indicate that the efficient electron transfer from CN to CNTs and further to NM obviously decreases the charge recombination. These are in accordance with the electrochemical impedance spectroscopy (EIS) and photocurrent density measurements. A single-particle PL study further confirms that with the addition of EG, the PL intensity of CN/CNTs/NM is significantly decreased because the photo-generated holes are captured by EG. Based on the above results, they propose the mechanism as follows: after the photo-excitation, the photo-generated electrons transfer from CN to CNTs and further to NM, where photo-generated electrons reduce protons to produce H_2_ gas. Photo-induced holes oxidize the EG to form glyoxal and carboxylate.

**Fig. 7:**
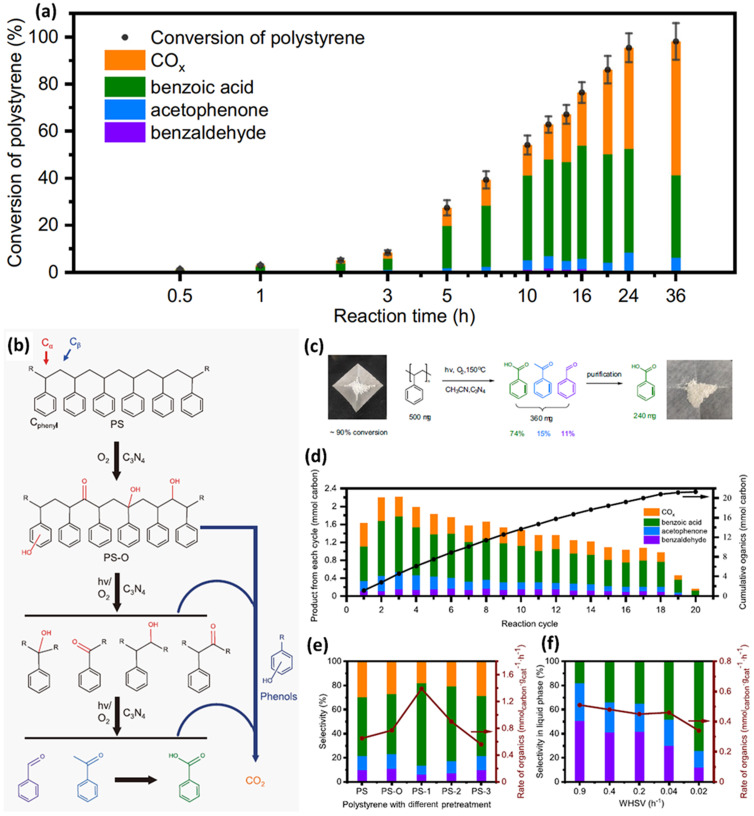
(a) Generation of various products for photocatalytic oxidation of PS. Reaction conditions: 10 mg PS (*M*_w_ = ∼50 kDa), 50 mg g-C_3_N_4_, 30 ml acetonitrile, 300 W xenon light, 150 °C and under 10 bar O_2_. Columns with various colours denote different generated products. The standard deviation of conversion in three parallel experiments were denoted by the error bars. (b) Proposed reaction pathways for photocatalytic oxidation of PS. (c) Schematic illustration for the conversion experiments of 500 mg PS pellets. (d) Photocatalytic oxidation of 500 mg PS pellets in 20 reaction cycles. Reaction conditions: 500 mg PS pellets, 200 mg g-C_3_N_4_, 40 ml acetonitrile, 150 °C, 10 bar O_2_ and 300 W xenon light illumination for 8 hours in each cycle. After every cycle, the solution is released and pure solvent is added. (e) Catalytic oxidation of PS after various pre-treatments. Reaction conditions: 20 mg PS, 50 mg g-C_3_N_4_, 30 ml acetonitrile, 150 °C and 300 W xenon light illumination for 8 hours. PS–O: thermal treatment at 150 °C in acetonitrile with O_2_. PS-1: thermal treatment at 220 °C in air. PS-2: thermal treatment at 300 °C in air. PS-3: pyrolysis at 350 °C in N_2_. (f) Performances of catalytic oxidation of PS at different weight hourly space velocities (WHSVs). Reaction conditions: 30 ml acetonitrile, 100 mg g-C_3_N_4_, 120 °C, 300 W xenon light irradiation, and a high pressure syringe pump used to pump PS solution (about 0.3 mg ml^−1^ in acetonitrile) into the reactor at different rates. 10 ml reaction solution was drained out manually as the PS solution pumped in amounted to the same volume. The standard deviation of conversion in 3 parallel experiments was denoted by the error bars. Reproduced with permission from ref. 44 Springer Nature.

Although it is very challenging to photo-reform untreated plastics at room temperature, in aqueous solution and under anaerobic conditions using C_*x*_N_*y*_, Cao *et al.*^44^ have successfully utilized C_3_N_4_ to photocatalytically convert PS into aromatic oxygenates, such as benzoic acid, acetophenone and benzaldehyde in acetonitrile at 80–150 °C, with light irradiation and in air. They have adopted various well-known, environmentally benign and simple-to-synthesize photocatalysts including TiO_2_, ZnO, ZnS and C_3_N_4_ to upcycle PS into aromatic oxygenates at 80 °C, with light illumination and in air. TiO_2_, ZnO, ZnS and C_3_N_4_ show a PS conversion of 13%, 21%, 12% and 55%, 64% and 60%, respectively. TiO_2_ shows the lowest selectivity of 15%, since the main products for TiO_2_ are CO_2_ and CO. This indicates that TiO_2_ is not a suitable photocatalyst for upcycling PS. Besides, despite that both ZnO and ZnS exhibit good selectivity, their PS conversion is not good. Thus, C_3_N_4_ was adopted. A range of metals, such as 0.1% Au, 0.5% Au, 0.5% Pt, 0.5% Fe and 0.5% Cu, were loaded on C_3_N_4,_ respectively. Although these metal loadings could increase the conversion to some extent, the selectivity was reduced, compared to C_3_N_4_ without metal loading. These might be boosted by the overoxidation of intermediates/products. Then, they have investigated the photocatalytic oxidation of PS using g-C_3_N_4_. The time-evolution experimental results (Fig. 7a) exhibit an obvious induction period in the first 3 hours, followed by the fast accumulation of various inorganic/organic products (CO_*x*_, benzoic acid, acetophenone and benzaldehyde) in 24 hours. Based on the spectroscopic result, gel permeation chromatography (GPC) and liquid product analysis, it was found that in the induction period of reaction, reactive oxygen species partially oxidize the PS. The reaction mechanism is disclosed in Fig. 7b as follows: (1) oxidative functionalization of PS with OH groups at C_α_ or C_phenyl_ sites as well as OH or C=O groups at C_β_ sites occurs under both thermo and light illumination conditions using C_3_N_4_; (2) with light illumination, C_3_N_4_ generates photo-excited electrons/holes to form ˙O_2_^−^ and possible carbon radical intermediates; (3) reactive oxygen radicals or oxidative photo-generated holes easily attack PS–O to generate the C–C–O˙ intermediate, resulting in the breakage of the C–C bond and scission of the polymer backbone *via* the β scission procedure. Overoxidation of carbon containing reactants/intermediates/products could occur in any of the above steps, thus generating undesired CO_2_. As shown in Fig. 7c, they can utilize this strategy to acquire pure chemicals, such as 240 mg benzoic acid, *via* using column chromatography separation. As presented in Fig. 7d, *via* appropriately regulating the ratio of substrate/catalyst (5 : 2) and reaction time (8 hours), they can acquire a stable yield rate of various organics (10 mg g^−1^ h^−1^) with a selectivity of 76% in 18 cycles for photocatalytic oxidative conversion of PET plastic pellets (500 mg). Furthermore, they utilized a flow reaction system to optimize the activity and selectivity towards specific products *via* tuning the weight hourly space velocity (WHSV). They found that better selectivity for benzaldehyde (51%) and acetophenone (31%) is acquired with an optimized WHSV of 0.9 h^−1^ (Fig. 7f).

The above four studies show the great potential of using C_*x*_N_*y*_ based photocatalysts to upcycle pre-treated polyester/polyolefin at room temperature and under anaerobic conditions or even untreated polyolefin at raised temperature and in air/O_2_. Owing to the significant advantages of earth-abundance, cost-effectiveness, excellent absorption of light and suitable redox abilities, further investigation on C_*x*_N_*y*_ based photocatalysts is anticipated. All the performances and reaction conditions of the studies in this section are summarized in Table 3.

**Table 3:** Non-metal based photocatalysts for photo-reforming of plastics

Non-metal based catalyst	Pre-treatment	Product after pre-treatment	Reaction conditions	Activity and stability	Reference (year)
Carbon nitride combined with a nickel phosphide (Ni_2_P) cocatalyst (CN_*x*_|Ni_2_P)	50 mg ml^−1^ PET soaked in 2 M aqueous KOH, 24 h, 40 °C	PET converted to ethylene glycol, terephthalate	Simulated solar light (AM 1.5G, 100 mW cm^−2^), 2 ml 1 M aqueous KOH, 1.6 mg ml^−1^ catalyst, N_2_ atmosphere, 25 °C	H_2_ (25.7 ± 2.3 μmol g^−1^ h^−1^), 50 h reaction acetate (190 nmol), formate (190 nmol), glyoxal (9300 nmol), 5 day reaction	41 (2019)
	50 mg ml^−1^ PLA soaked in 2 M aqueous KOH, 24 h, 40 °C	PLA converted to lactate		H_2_ (55.7 ± 3.7 μmol g^−1^ h^−1^), 50 h reaction acetate (100 nmol), formate (95 nmol), 5 day reaction	
Carbon nitride combined with a nickel phosphide (Ni_2_P) cocatalyst (CN_*x*_|Ni_2_P)	25 mg ml^−1^ PET soaked in 0.5 M aqueous KOH, 80 °C, overnight, in air	—	Solar simulator (AM 1.5G, 100 mW cm^−2^), 2 ml min^−1^ flow rate, 50 ml 25 mg ml^−1^ pre-treated PET solution, 25 cm^2^ catalyst panel, 25 °C, N_2_ atmosphere	H_2_ (52 μmol m^−2^ h^−1^)	42 (2021)
Carbon nitride–carbon nanotubes–NiMo hybrid (CN–CNT–NiMo)	50 mg ml^−1^ PET soaked in 5 M KOH at 70 °C for 24 h	PET converted to ethylene glycol terephthalate and isophthalic acid	500 W Xe lamp (simulated solar light, 95 mW cm^−2^), 10 mg catalyst, 10 ml pre-treated PET aqueous solution, argon atmosphere	H_2_ (90 μmol g^−1^ h^−1^) using 10 M KOH aqueous solution	43 (2022)
g-C_3_N_4_	—	—	300 W xenon lamp, 50 mg catalyst, 20 mg PS, 30 ml acetonitrile solvent, 1 bar air, 150 °C	Selectivity benzoic acid (39%), acetophenone (7%), benzaldehyde (2%), CO_*x*_ (52%), conversion (95 ± 5%), 24 h reaction	44 (2022)

### 3.4 Composite photocatalysts for plastic upcycling

Currently, the reported composite photocatalysts for plastic upcycling are based on two photon absorbers, both of which can absorb photons and generate photo-induced electrons/holes for upcycling plastics. The effectiveness of composite photocatalysts is determined by the junction type and internal interaction between the components: (i) the most common type II heterojunction can increase light absorption and boost charge separation, but the redox abilities are compromised; (ii) Z-scheme heterojunctions can enhance light harvesting, accelerate electron–hole dissociation and reserve strong redox capabilities, simultaneously; (iii) strong and intimate interaction between different components, which are connected by chemical bonds rather than physical bonds, can significantly improve the charge separation efficiency and stability of overall composite photocatalysts, thus leading to greatly increased performances. The following five studies^45–49^ are categorized into two kinds of composite photocatalysts: (i) inorganic composite photocatalysts^45^ and (ii) inorganic/organic composite photocatalysts.^46–49^ An inorganic composite photocatalyst reported is a 0.5 wt% Pt loaded CdO_*x*_/CdS/SiC photocatalyst (Pt-CdO_*x*_/CdS/SiC).^45^ The as-synthesized Pt-CdO_*x*_/CdS/SiC was adopted to photo-reform various organic wastes (*e.g.*, PE, PS, IR, cellulose, lignin, albumin and keratin) in 10 M NaOH aqueous solution at 70 °C. The photocatalytic H_2_ evolution activities of 25.0, 19.4 and 36.7 μmol g^−1^ h^−1^ were observed in the presence of PE, PS and IR, respectively. It was found the raised temperature and increased basicity of the reaction system could significantly increase the photocatalytic H_2_ evolution of Pt-CdO_*x*_/CdS/SiC in the presence of α-cellulose, albumin or PE. The photocatalytic stabilities for reforming cellulose, albumin and PE were tested, respectively. Pt-CdO_*x*_/CdS/SiC exhibits good stability for reforming cellulose and albumin after re-addition of the substrate. In comparison, Pt-CdO_*x*_/CdS/SiC shows poor stability for reforming of PE even after re-addition of the substrate, probably because PE gels cover the photocatalysts at raised temperature. The increased photocatalytic performance of CdO_*x*_/CdS/SiC is attributed to the formation of a type II heterojunction between CdS and SiC resulting in enhanced charge separation/transfer.

**Fig. 8:**
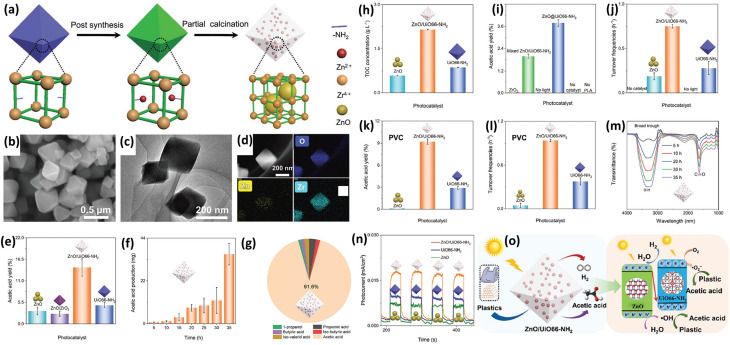
(a) Schematic image showing the synthesis of ZnO/UiO66-NH_2_ using post-synthesis and partial calcination. (b) SEM image and (c) low magnification TEM images of UiO66-NH_2_. (d) HAADF-STEM image of the as-synthesized ZnO/UiO66-NH_2_ and corresponding elemental mapping images. (e) Photocatalytic yields of acetic acid for ZnO, ZnO/ZrO_2_, UiO66-NH_2_ and ZnO/UiO66-NH_2_. (f) Generation of acetic acid for ZnO/UiO66-NH_2_ at different reaction times. (g) Selectivity of various products for ZnO/UiO66-NH_2_. (h) Concentration of TOC for ZnO, ZnO/UiO66-NH_2_ and UiO66-NH_2_ reaction systems. (i) Yields of acetic acid on ZrO_2_, mixed ZnO/UiO66-NH2, and ZnO/UiO66-NH_2_, without light, without a catalyst or without PLA. (j) TOF for H_2_ evolution on UiO66-NH_2_, ZnO or ZnO/UiO66-NH_2_ in PLA reaction systems. (k) Yield of acetic acid and (l) TOF for H_2_ evolution on ZnO, ZnO/UiO66-NH_2_ or UiO66-NH_2_ in PVC reaction systems. (m) FTIR spectra of reaction solution in photocatalytic reforming of PLA using ZnO/UiO66-NH_2_ at different reaction times. (n) Transient photocurrent density measurements for ZnO, UiO66-NH_2_ and ZnO/UiO66-NH_2_. (o) Schematic image for the photo-generated charge dissociation/transfer in ZnO/UiO66-NH_2_. Reproduced with permission from ref. 47 American Chemical Society.

Reported inorganic/organic composite photocatalysts are categorized into inorganic/MOF based composite photocatalysts^46,47^ and inorganic/C_3_N_4_ based composite photocatalysts,^48,49^ respectively. The Zhang group have reported two research studies on inorganic/MOF based composite photocatalysts.^46,47^ The Zhang group^47^ have synthesized a zinc oxide (ZnO)/UiO66-NH_2_ composite *via* a partial calcination route, with ultra-small-sized ZnO nanoparticles (NPs) confined into the framework of UiO66-NH_2_. The as-synthesized ZnO/UiO66-NH_2_ composite photocatalyst is utilized for photocatalytic valorisation of PLA and PVC. First, they utilized a post-synthesis route to acquire Zn-UiO66-NH_2_*via* coordinating Zn^2+^ with the –NH_2_ group in Zn-UiO66-NH_2_ (Fig. 8a). Then, ZnO/UiO66-NH_2_ with a porous structure was acquired *via* annealing Zn-UiO66-NH_2_ in air at 350 °C (Fig. 8a). The SEM image of ZnO/UiO66-NH_2_ exhibits a uniform rhombic octahedral morphology exposed with a smooth surface (Fig. 8b), indicating that the raw structure of UiO66-NH_2_ is reserved after the synthesis. The TEM image of ZnO/UiO66-NH_2_ shows a uniform particle size of about 200 nm (Fig. 8c). The STEM-HAADF image and corresponding elemental mapping images of ZnO/UiO66-NH_2_ (Fig. 8d) show the homogeneous distribution of O, Zn and Zr elements on ZnO/UiO66-NH_2_. As for PLA valorisation, the ZnO/UiO66-NH_2_ composite exhibits a higher acetic acid yield of 14.4%, compared to those of ZnO (3.3%) and UiO66-NH_2_ (4.7%), respectively (Fig. 8e). They found that the acetic acid generation in the preliminary stage is low followed by gradual enhancement with increasing time (Fig. 8f). This is attributed to the small exposed surface area and low hydrophilicity of big-sized PLA particles in the beginning. Nevertheless, as reaction time increases, these big-sized PLA particles are gradually transformed into small-sized PLA particles with increased hydrophilicity, leading to a larger exposed surface area and higher activity. The ZnO/UiO66-NH_2_ composite shows an excellent selectivity of 91.6% for acetic acid *via* valorisation of PLA (Fig. 8g). As for the control experiments, the ZnO/UiO66-NH_2_ composite shows a much higher acetic acid yield (14.4%) than ZnO (3.3%) and physically mixed ZnO/UiO66-NH_2_ (2.0%), respectively. These results indicate the great importance of intimate interaction between ZnO and UiO66-NH_2_. Fig. 8h exhibits the total organic carbon (TOC) concentrations of ZnO/UiO66-NH_2_ (2.1 g l^−1^), ZnO (0.6 g l^−1^) and UiO66-NH_2_ (0.9 g l^−1^). The obviously larger TOC concentration for ZnO/UiO66-NH_2_ suggests its capability of continuously transforming PLA into soluble organic chemicals, *e.g.*, acetic acid, boosted by the synergistic effect between UiO66-NH_2_ and ZnO. Control experiments (Fig. 8i) exhibit much lower acetic acid generation for physically mixed ZnO/UiO66-NH_2_ (2.0%) and negligible acetic acid generation without light, a catalyst or PLA, suggesting the key role of strong interaction between ZnO and UiO66-NH_2_. Further study shows that no acetic acid was detected as a N_2_ atmosphere is adopted for photocatalytic PLA conversion, indicating the key role of O_2_ in photocatalytic plastic conversion. Additionally, ZnO/UiO66-NH_2_ also exhibits excellent stability for photocatalytic PLA valorisation with no obvious change observed on the compositions, structures and morphologies. Besides, ZnO/UiO66-NH_2_ also exhibits the generation of H_2_ in photocatalytic PLA valorisation, with the TON (26.36) and TOF (0.75 h^−1^) observed for H_2_ evolution (Fig. 8j). The ZnO/UiO66-NH_2_ composite was also utilized for photocatalytic PVC valorisation, with an acetic acid yield of 9.2% (Fig. 8k) as well as TON of 33.13 and TOF of 0.95 h^−1^ for H_2_ evolution (Fig. 8l). Compared to ZnO and UiO66-NH_2_, the increased photocatalytic activity of the ZnO/UiO66-NH_2_ composite arises from the broad light absorption, rapid charge dissociation/migration and highly exposed active sites. Then, they use FTIR spectroscopy to test the intermediate products in PLA valorisation by the ZnO/UiO66-NH_2_ composite. As time increases, the rising intensities of two peaks at 1760 and 3400 cm^−1^, ascribed to the C=O and –OH of the carboxylic acid, respectively, are observed (Fig. 8m). These results also confirm the capability of the ZnO/UiO66-NH_2_ composite to transform PLA into carboxylic acid containing substances in photocatalysis. Furthermore, they also confirm that the developed ZnO/UiO66-NH_2_ composite could convert LDPE and PET through a photocatalysis reaction. They also demonstrate that the ZnO/UiO66-NH_2_ composite can valorise a commercial PLA bag and PLA straw *via* photocatalysis. Photoelectrochemical (PEC) current density measurement (Fig. 8n) shows the largest PEC current density for ZnO/UiO66-NH_2_, again confirming the key role of intimate interaction between ZnO and UiO66-NH_2_. Based on the above results, the excellent PLA conversion of the ZnO/UiO66-NH_2_ composite is attributed to the following reasons: (i) partial annealing route reserves the highly porous structure of ZnO/UiO66-NH_2_, thus supplying numerous active centres; (ii) the combination of porous UiO66-NH_2_ and ZnO leads to efficient interfacial charge separation/migration; (iii) the combination of ZnO with UiO66-NH_2_ optimises the electronic structure. ESR results and quenching experiments together reveal that the radicals of ˙O_2_^−^ and ˙OH together play a key role in photocatalytic PLA conversion (Fig. 8o). Besides, Z-scheme charge separation/transfer is increased for the ZnO/UiO66-NH_2_ composite (Fig. 8o). Finally, a possible reaction pathway is proposed for photocatalytic PLA conversion, and active radicals preferentially cleave the C–O bond of the PLA chain in photocatalysis, leading to the gradual cracking of the PLA carbon chain. Then, PLA is transformed into PLA plastic fragments or oligomers by these active radicals. At last, these PLA plastic fragments or oligomers are transformed into acetic acid by the ZnO/UiO66-NH_2_ composite. For the conversion of PVC, they propose that the active radicals first cleave the C–Cl bond, followed by further oxidation to oxygen-containing organic intermediates released into the reaction solution. Finally, active radicals further oxidize these organic intermediates to generate acetic acid. In another study, the Zhang group^47^ have fabricated Ag_2_O NPs embedded in an Fe-based MOF *via in situ* conversion of unstable Ag sites in a Fe–Ag bimetallic MOF. First, they synthesized the Fe-based MOF followed by a post-synthesis technique to acquire the Fe–Ag bimetallic MOF. Then, the as-synthesized Fe–Ag bimetallic MOF was subjected to light irradiation to form the Ag_2_O NP enclosed Fe-based MOF, denoted as Ag_2_O/Fe-MOF. The MOF structure impedes the growth of Ag_2_O NPs and renders better dispersion of Ag_2_O NPs. XRD results confirm that the addition of Ag sites and Ag_2_O generates defects in the structure of the Fe based MOF. XPS results reveal strong electronic coupling between added Ag sites and the Fe-based MOF in the Fe–Ag bimetallic MOF. XPS results also disclose the defects generated in the structure of the Fe–Ag bimetallic MOF and Ag_2_O/Fe-MOF. The XPS results also indicate the oxygen vacancies formed in Ag_2_O/Fe-MOF, due to the cleaving of Fe–O bonds following light illumination. Besides, the etching XPS test of Ag_2_O/Fe-MOF shows the increasing peak intensity as the etching time increases, further confirming that Ag_2_O NPs are incorporated into the pores of the Fe based MOF. The incorporation of Ag_2_O NPs in the pores of Fe-MOF is also confirmed by the N_2_ sorption analyses, which reveal the obviously reduced surface area of Ag_2_O/Fe-MOF (110 m^2^ g^−1^) compared with that of the Fe–Ag bimetallic MOF (268 m^2^ g^−1^). Nevertheless, this surface area of Ag_2_O/Fe-MOF (110 m^2^ g^−1^) is still much higher than that of bare Ag_2_O (17 m^2^ g^−1^), highlighting the advantage of the structure for Ag_2_O/Fe-MOF. As the porous Ag_2_O/Fe-MOF can supply a much larger surface area with increased active centres, compared with the Fe based MOF, Ag_2_O/Fe-MOF exhibits an obviously widened light absorption range, which will boost the photocatalytic performance. Furthermore, MS plots further indicate that the Fe based MOF and Ag_2_O are p-type and n-type semiconductors, respectively. These results reveal the formation of a p–n junction in Ag_2_O/Fe-MOF. Then, transient photocurrent (TPC) density measurements were conducted on bare Ag_2_O, the Fe based MOF and Ag_2_O/Fe-MOF. The results show that the TPC density values are as follows: Ag_2_O/Fe-MOF > Fe-MOF > Ag_2_O. The highest TPC density value of Ag_2_O/Fe-MOF is attributed to the formation of a p–n junction in Ag_2_O/Fe-MOF, leading to increased dissociation/transportation of electrons/holes. Besides, the existence of defects in the structures of Ag_2_O/Fe-MOF leads to more open frameworks and active centres, thus inducing enhanced photo-generated electrons/holes. Additionally, the results of EIS, steady-state PL spectroscopy and bode-phase plots also confirm the highest efficiency of charge separation/transfer for Ag_2_O/Fe-MOF among these samples, in accordance with the TPC density measurement results. Then, the as-synthesized Ag_2_O/Fe-MOF is adopted for photocatalytic H_2_ evolution coupled with upcycling of PEG. With the introduction of 0.2 wt% Ag_2_O in Fe-MOF, Ag_2_O/Fe-MOF (0.2 wt%) exhibits the highest photocatalytic PET MP weight loss (27.5 mg in 3 hours) and H_2_ evolution (6.2 mmol g^−1^ in 2.5 hours). In contrast, with lower (0.05 wt%) or higher (1 wt%) Ag_2_O NPs introduced, both Ag_2_O/Fe-MOF (0.05 wt%) and Ag_2_O/Fe-MOF (1 wt%) exhibit inferior photocatalytic PET MP weight loss and H_2_ evolution. This is because the introduction of Ag_2_O NPs could boost the catalytic activity of Ag_2_O/Fe-MOF, whilst excessively introduced Ag_2_O NPs induce some destruction in the structure of Fe-MOF, thus reducing the number of active sites. In contrast, bare Ag_2_O and the Fe MOF exhibit inferior PEG conversion efficiency owing to their unfavourable band gap and insufficient light absorption. Additionally, Ag_2_O shows no H_2_ evolution since the CB of Ag_2_O (0.12 V *vs.* NHE) is lower than the H_2_ evolution potential (0 V *vs.* NHE). The physically mixed sample of Ag_2_O and Fe-MOF (Ag_2_O@Fe-MOF) exhibit a low PEG conversion (6.1 mg) and H_2_ evolution (2.3 mmol g^−1^). These results reveal that the developed photochemistry route induces intimate interaction between Ag_2_O and Fe-MOF for efficient charge transport. The small sizes of Ag_2_O NPs incorporated in the pores of Fe-MOF ensure the exposure of abundant active sites, leading to enhanced conversion of MPs. Total organic carbon (TOC) analysis results disclose a remarkable increase in TOC for Ag_2_O/Fe-MOF and Fe-MOF, since the photocatalyst starts to transform the MPs into water-dissolvable long-chain fragments. This also results in the obvious reduction of the weight on PEG in the beginning of photocatalysis. Further analysis indicates the formation of small amounts of acetic acid in 0–5 hours of photocatalytic reaction, suggesting that in the beginning of photocatalysis radicals convert PEG MPs into long-chain fragments. The generation of ethanol and formic acid is also disclosed. The increased TOC concentration is much higher than the formation of formic acid, ethanol and acetic acid in total, indicating that some of the PEG MPs were transformed into soluble MPs and other intermediates. They also tested the photocatalytic reforming of PE and PET MPs for all the samples. Ag_2_O/Fe-MOF exhibits much higher PE and PET MP weight loss, compared with bare Ag_2_O and Fe-MOF, respectively. Ag_2_O/Fe-MOF also exhibits the increased photocatalytic H_2_ evolution activities of 1.7 and 1.9 mmol g^−1^ h^−1^ for PE and PET MPs, respectively. The above results not only corroborate that the incorporation of Ag_2_O into porous Fe-MOF can be adopted for photocatalytic reforming of PEG/PE/PET MPs, but also confirm that the active centres arising from structure defects can boost the plastic upcycling. The robustness of the activities and structures for Ag_2_O/Fe-MOF is also confirmed. ESR experiments confirm the existence of ˙OH radicals from Ag_2_O/Fe-MOF in photocatalysis. And they propose a possible reaction pathway: PEG → ethylene glycol → glycolaldehyde → glycolate → glyoxylate → acetic acid → formic acid. Polyoxometalates (POMs), or transition metal oxygen anion clusters, are selected since they show outstanding robustness unaffected by pH, solvent and temperature. Besides, these POMs exhibit reversible multi-electron redox conversions while reserving robust structures, leading to their wide application in photo-/electro-catalysis fields. Especially, phosphovanadomolybdate (H_5_PMo_12−*n*_VnO_40_) is extensively adopted as the active catalyst involving in organic oxidation with O_2_. More importantly, vanadium atoms with variable valence in Keggin-structured H_5_PMo_10_V_2_O_40_ exhibits the capability for catalysing C–C bond cleavage reaction *via* the electron transfer-oxygen transfer reaction. Nevertheless, the rapid recombination of photo-induced electrons/holes and low redox capabilities for VPOM, as decided by its bandgap structure, significantly impedes its application in photocatalysis. Thus, it is desirable to combine VPOM with other materials for constructing VPOM based composite photocatalysts, thus achieving enhanced charge separation/transfer efficiency and increased catalytic abilities.

The other two studies are based on inorganic/polymerized C_3_N_4_ based composite photocatalysts.^48,49^ One work^48^ reports a heterostructure composed of V-substituted phosphomolybdic acid clusters coupled with g-C_3_N_4_ nanosheets (VPOM/CNNS). The FTIR spectra of VPOM/CNNS exhibit distinctive vibration modes of CNNS and Keggin units of VPOM, confirming the successful combination of CNNS with VPOM. Besides, VPOM/CNNS also shows a similar surface area to CNNS (103.51 m^2^ g^−1^), suggesting that CNNS reserving its ultrathin nanosheet morphology after combining with VPOM clusters. TEM results show that the VPOM/CNNS composite reserves the distinct two-dimensional layered structure. Aberration-corrected high-angle annular dark field scanning transmission electron microscopy (AC-HAADF-STEM) combined with elemental mapping analysis confirms the uniform distribution of VPOM clusters on the surface of CNNS. The XPS results confirm the electron transfer from CNNS to VPOM in the VPOM/CNNS composite. The newly formed peaks in the XPS O 1 s spectra indicate the generation of C–O–Mo or C–O–V bonds, again revealing the coupling of VPOM with CNNS. VPOM/CNNS exhibits increased light harvesting in the range of 460–600 nm, in contrast with bare CNNS, again revealing the existence of VPOM in VPOM/CNNS. *Via* combining the XPS VB and UPS results, they found that VPOM and CNNS construct a type II hetero-junction with a built-in electric field pointing from CNNS to VPOM. Furthermore, *in situ* XPS results confirm the accumulation of photo-induced electrons and holes in CNNS and VPOM, respectively, with light illumination. These results confirm the Z-scheme charge transfer in the VPOM/CNNS composite. Femtosecond transient absorption spectroscopy (fs-TAS) was adopted to study the photo-induced electron/hole kinetics in VPOM/CNNS. *Via* applying AgNO_3_ as the electron scavenger, they found that the peak at ∼686 nm is ascribed to the CNNS˙^−^ absorption and the signal at 550 nm is principally attributed to the photo-induced holes of CNNS. VPOM/CNNS exhibits increased CNNS˙^−^ absorption at ∼686 nm, compared to bare CNNS, suggesting the more effective separation/transfer of photo-generated electrons/holes. Furthermore, they found an additional decay component (*τ*_3_ = 19.42 ps) for the hole species of VPOM/CNNS, which is ascribed to the Z-scheme charge transfer pathway in the heterostructure interface. VPOM/CNNS composites all exhibit increased decay lifetimes of electron species, compared to CNNS alone, suggesting the more efficient dissociation/migration of photo-generated electrons in VPOM/CNNS. ESR experiments further show the obvious enhanced signals of DMPO-˙O_2_^−^ and DMPO-˙OH signals compared with CNNS or VPOM alone, again confirming the increased charge kinetics in the Z-scheme junction. Then, the as-synthesized photocatalysts were adopted for photocatalytic reforming of a range of plastics. First, they were utilized to photo-reform PE as it is extensively applied and not biodegradable. The optimised VPOM-CNNS composite shows an outstanding photocatalytic HCOOH generation rate (24.66 μmol h^−1^ g^−1^), about 262 times larger than that of CNNS alone. The optimised VPOM-CNNS composite also exhibits better photocatalytic activity than the mechanically mixed VPOM and CNNS. These further corroborate that the intimate interaction between VPOM and CNNS could obviously increase the charge separation/transfer efficiency. Additionally, a 100 hour stability test was also conducted on the VPOM/CNNS composite. Excellent stability of HCOOH generation was observed on the VPOM/CNNS composite for photocatalytic reforming of PE. After a 100 hour reaction, no apparent alteration can be found in the composition/structure of the VPOM/CNNS composite. Besides, VPOM/CNNS was also used for photocatalytic reforming of PP, PVC, PEG and PAM. HCOOH is identified as the upcycled product. The HCOOH generation rate from photocatalytic upcycling of PEG and PAM is much larger than that of the others. The reasons are as follows: (i) the polarity groups in PEG (hydroxyl and ether groups) and polyacrylamide (–NH_2_ group) significantly facilitate their dissolution in polar solvents of acetonitrile/water, thus increasing the possibility for the plastic molecules to react with the photo-generated active species; (ii) it is much easier to activate the asymmetric C–O bonds in the PEG, compared to the inert nonpolar C–C bonds. Additionally, VPOM/CNNS also exhibits outstanding photocatalytic HCOOH generation from photocatalytic reforming of real-word PE bags, PVC plastic wrap or PP surgical masks. Species-trapping experiments were also carried out *via* using nitrogen, *p*-benzoquinone, oxalic acid and nitrobenzene as the O_2_, ˙O_2_^−^, h^+^ and e^−^ scavengers, respectively. H^+^ and ˙O_2_^−^ were identified as the principal reactive species towards photocatalytic reforming of plastics. ^1^H NMR spectroscopy was further adopted to study the photocatalytic reforming reaction. After 36 hour visible-light illumination, apart from HCOOH as the principal product, large amounts of long-chain alcohols and a trace amount of formaldehyde were identified in the liquid phase. These are common intermediates in the electron transfer-oxygen transfer oxidation reaction of vanadium compounds. This is further confirmed by the observation of an eight-line signal of V^IV^ after light illumination in an argon atmosphere, suggesting that some of the V^V^ species in VPOM are reduced to form V^IV^ in the photocatalytic reforming of PE reaction. IR spectra reveal the generation of new carbonyl groups in the range of 1710–1760 cm^−1^ in photocatalytic reforming of PE plastic bags. And the generation of peroxides was also confirmed to arise from the reaction between alkyl radicals and reactive oxygen species. DFT computations were also conducted to acquire the insightful understanding. The computation results indicate that the C and N elements in CNNS and O elements in VPOM serve as the principal reactive sites towards photocatalytic reforming of plastics. On the basis of all the results, they propose a photocatalytic mechanism: with visible-light illumination, both VPOM and CNNS will be excited to generate abundant photo-induced electrons and holes. *Via* the ligand to metal charge transfer (LMCT), the electrons from the O atom in the HOMO of VPOM is excited to an antibonding orbital of the LUMO in the transition metal centres (V or Mo). For CNNS, with light illumination, electrons are excited from the HOMO or N 2p states to the LUMO or hybridized C 2p and N 2p states. Then, the photo-induced electrons and holes will migrate and dissociate following the Z-scheme scheme. Afterwards, the photo-induced holes remaining in the HOMO (O 2p states) of VPOM will from oxo-centred radicals. Finally, the highly active and photo-excited VPOM clusters would boost the oxidative cleavage of the C–C bond, leading to the formation of formaldehyde and a carbon-centred alkyl radical. Then, reactive oxygen species will oxidize the alkyl radical to generate alkyl peroxide groups, which is transformed into long-chain alcohols. At the same time, formaldehyde will be oxidized to generate formic acid, by the generated ˙O_2_^−^. Furthermore, Gong *et al.*^49^ have developed a metal-free photocatalyst composed of carbonized polymer dot-graphitic carbon nitride (CPDs-CN). The as-synthesized carbonized polymer dots (CPDs) possess a big conjugated graphitic sp^2^ carbon combined with sp^3^ carbons, as confirmed by ^1^H and ^13^C NMR spectroscopy. FTIR and XPS spectroscopy techniques together confirm the existence of carboxylic, hydroxyl and amino functional groups in CPDs. The TEM images show the CPDs-CN composite comprising CPDs with sizes of 1.9–2.4 nm loaded on the surface of CN sheets. The XPS results confirm the combination of CPDs with CN sheets *via* forming amide bonds. The coupling of CPDs with CN sheets also leads to the change of colour from light yellow for CN to dark brown for CPDs-CN, thus increasing the light absorption in the whole visible-light range (400–800 nm). The as-synthesized CPDs-CN was utilized for photocatalytic reforming of PET and PLA. Photocatalytic reforming of the pre-treated PET solution leads to generation of abundant EG-derived chemicals, such as glycolaldehyde, glycolic acid, formic acid, ethanol, acetaldehyde and acetic acid after 8 day photocatalytic reforming using CPDs-CN. ^1^H and ^13^C NMR spectroscopy reveal that: (i) PET plastic conversion is increases monotonically with increasing time; (ii) high selectivity is achieved for glycolic acid and acetic acid; (iii) little selectivity change is observed for the intermediates, such as glycolaldehyde, formic acid, ethanol and acetaldehyde. The reaction pathway is revealed: photo-induced holes first oxidize EG to form glycolaldehyde, followed by further oxidation to generate glycolic acid and formic acid. Besides, EG could also be dehydroxylated to generate ethanol, followed by further oxidation to acetaldehyde and acetic acid. The conventional anatase TiO_2_ exhibits inferior photocatalytic activities for generating the above chemicals, compared to CPDs-CN. The photocatalytic H_2_ evolution activities coupled with PET/PLA hydrolysis were also determined. Without Pt as the co-catalyst, CPDs-CN exhibits a photocatalytic H_2_ evolution activity of 298 ± 58 μmol g^−1^ h^−1^ using pre-treated PET as the substrate. In comparison, with Pt loaded as the co-catalyst, CPDs-CN exhibits a photocatalytic H_2_ evolution activity of 1034 ± 134 and 1326 ± 181 μmol g^−1^ h^−1^*via* using pre-treated PET and PLA as the substrate, respectively. As a contrast, anatase TiO_2_ loaded with Pt only exhibits a photocatalytic H_2_ evolution activity of 55 ± 4 μmol g^−1^ h^−1^ using pre-treated PET as the substrate. The experimental results show that CPDs can obviously increase the light harvesting and boost the dissociation/transportation of photo-excited electrons/holes, thus leading to the increased activities of CPDs/CN. Also, no ˙OH was detected *via* the fluorescence experiment, suggesting that photo-excited holes play a key role in oxidation of substrates.

The above five studies underscore the appealing prospects of composite photocatalysts for plastic upcycling, which exhibit both an extended absorption range of light and efficient charge separation/transfer.^45–49^ Especially, MOF based composite photocatalysts show excellent performances for upcycling untreated polyesters/polyolefins in air and in organic solvent (*e.g.*, acetonitrile).^46,47^ Besides, poly-oxalate based composite photocatalysts also exhibit outstanding activities/selectivity for upcycling untreated polyesters/polyolefins in an O_2_ atmosphere and in organic solvent (*e.g.*, acetonitrile).^48^ All the performances and reaction conditions in this section are summarized in Table 4.

**Table 4:** Composite photocatalysts for photo-reforming of plastics

Composite catalyst	Pre-treatment	Products after pre-treatment	Reaction conditions	Activity and stability	Reference (year)
0.5 wt% Pt-CdO_*x*_/CdS/SiC	—	—	Solar simulator (AM 1.5G), 50 mg catalyst, 5 ml 10 M NaOH aqueous solution, 100 mg PE, AR atmosphere, 70 °C	H_2_ (25.0 μmol g^−1^ h^−1^)	45 (2022)
	—	—	Solar simulator (AM 1.5G), 50 mg catalyst, 5 ml 10 M NaOH aqueous solution, 100 mg PE, AR atmosphere, 70 °C	H_2_ (19.4 μmol g^−1^ h^−1^)	
ZnO/UiO66-NH_2_	—	—	300 W Xe lamp, 0.1 g catalyst, 1.0 g PLA, 50 ml water, in air, 25 °C	PLA conversion rate (57.1 mg g^−1^ h^−1^), acetic acid evolution (selectivity = 91.6%; yield = 14.4%; TON = 17.92; TOF = 0.51 h^−1^), H_2_ evolution (TON = 26.36; TOF = 0.75 h^−1^)	46 (2022)
	—	—	300 W Xe lamp, 0.1 g catalyst, 1.0 g PVC, 50 ml water, in air, 25 °C	PVC conversion rate (21.4 mg g^−1^ h^−1^), acetic acid evolution (yield = 9.2%; TON = 0.90; TOF = 0.03 h^−1^), H_2_ evolution (TON = 33.13 and TOF = 0.95 h^−1^)	
Ag_2_O nanoparticle encapsulated Fe based MOF (Ag_2_O/Fe-MOF)	—	—	300 W Xe lamp (AM 1.5G, 100 mW cm^−2^), 0.1 g catalyst, 0.5 g PEG MPs, 100 ml water, 25 °C, in air	H_2_ (3.6 mmol g^−1^), 2.5 h reaction, acetic acid (11.7 mg l^−1^), 5 h reaction	47 (2023)
	10 g PET immersed in 1 M NaOH solution at 65 °C, stirred for two weeks followed by centrifuging and freeze-drying the undissolved plastic pieces	PET MPs	300 W Xe lamp (AM 1.5G, 100 mW cm^−2^), 0.1 g catalyst, 0.5 g PET MPs, 100 ml water, 25 °C, in air	H_2_ (1.9 mmol g^−1^ h^−1^)	
	10 g PE immersed in 1 M NaOH solution at 65 °C, stirred for two weeks followed by centrifuging and freeze-drying the undissolved plastic pieces	PE MPs	300 W Xe lamp (AM 1.5G, 100 mW cm^−2^), 0.1 g catalyst, 0.5 g PE MPs, 100 ml water, 25 °C, in air	H_2_ (1.7 mmol g^−1^ h^−1^)	
V-Substituted phosphomolybdic acid clusters/g-C_3_N_4_ nanosheets (VPOM/CNNS)	—	—	300 W Xe lamp with a 420 nm cut-off optical filter, 10 mg catalyst, 20 mg PE, 10 ml acetonitrile, O_2_ atmosphere, 20–40 °C	Formic acid evolution (24.66 μmol g^−1^ h^−1^), 36 h reaction	48 (2021)
	—	—	300 W Xe lamp with a 420 nm cut-off optical filter, 10 mg catalyst, 20 mg PEG, 10 ml acetonitrile, O_2_ atmosphere, 20–40 °C	Formic acid evolution (208.65 μmol g^−1^ h^−1^), 36 h reaction	
	—	—	300 W Xe lamp with a 420 nm cut-off optical filter, 10 mg catalyst, 20 mg PP, 10 ml acetonitrile, O_2_ atmosphere, 20–40 °C	Formic acid evolution (26.68 μmol g^−1^ h^−1^), 36 h reaction	
	—	—	300 W Xe lamp with a 420 nm cut-off optical filter, 10 mg catalyst, 20 mg PVC, 10 ml acetonitrile, O_2_ atmosphere, 20–40 °C	Formic acid evolution (29.85 μmol g^−1^ h^−1^), 36 h reaction	
	—	—	300 W Xe lamp with a 420 nm cut-off optical filter, 10 mg catalyst, 20 mg PAA, 10 ml water, O_2_ atmosphere, 20–40 °C	Formic acid evolution (156.57 μmol g^−1^ h^−1^), 36 h reaction	
Carbonized polymer dot coupled graphitic carbon nitride (CPD-CN)	Refluxing PET in 5 M KOH aqueous solution at 120 °C for 24 h	PET converted to ethylene glycol terephthalic acid and isophthalic acid	300 W xenon lamp (AM 1.5G), 20 mg catalyst, 1.25 g PET, 50 ml 1 M KOH aqueous solution, 40 °C, vacuum environment	H_2_ (1034 ± 134 μmol g^−1^ h^−1^), selectivity, glycolaldehyde (4%), glycolic acid (21%), formic acid (12%), ethanol (37%), acetaldehyde (12%), acetic acid (14%)	49 (2022)
	Refluxing PLA in 5 M KOH aqueous solution at 100 °C for 2 h	PLA converted to lactate	—	H_2_ (1326 ± 181 μmol g^−1^ h^−1^)	

## 4. Conclusion and outlook

The above studies introduce the current achievements in various photocatalysts for plastic upcycling. These photocatalysts were categorized into four different types: (i) metal oxide based photocatalysts; (ii) metal sulphide based photocatalysts; (iii) non-metal based photocatalysts and (iv) composite photocatalysts. Usually, metal oxide based photocatalysts (*e.g.*, TiO_2_) possess very positive valence band potentials and strongly oxidative photo-excited holes, which can generate highly oxidative ˙OH radicals to directly oxidize the inert/robust plastics (*e.g.*, PE, PP and PVC) and cleave the strong C–C/C–O/C–H bonds to form value-added short-chain chemicals/fuels under aerobic/anaerobic conditions and at room temperature. In some work, metal oxide based photocatalysts, *e.g.* Nb_2_O_5_, can even fully oxidize the inert polyolefins (*e.g.*, PE, PP and PVC) to yield CO_2_ in air and at room temperature, which can be further reduced to form a C_2_ chemical (acetic acid). But the evolution efficiencies of these chemicals/fuels for metal oxide based photocatalysts are restricted owing to the wide bandgap width and weak absorption of solar light. In contrast, metal sulphide based photocatalysts (*e.g.*, CdS/CdO_*x*_ and Cd_0.5_Zn_0.5_S) with suitable bandgap widths exhibit strong absorption of light and favourable redox abilities. Photo-induced holes of metal sulphide based photocatalysts possess moderate oxidation abilities to upcycle the pre-treated polyester/polyolefin solutions and yield value-added chemicals without their overoxidation to form CO_2_. On the other hand, photo-excited electrons of metal sulphide based photocatalysts with sufficient reduction capacity could efficiently yield H_2_ fuel under anaerobic conditions. Even though metal sulphide based photocatalysts exhibit the above advantages, the insufficient stability and notorious toxicity, especially for Cd-based photocatalysts, significantly restrict their industrial scale applications for solar plastic upcycling. Furthermore, non-metal based photocatalysts, *e.g.*, C_*x*_N_*y*_ based catalysts, have shown many attractive edges, such as cheapness, high abundance, suitable bandgap width, adequate redox capacities, strong robustness against photo-/chemical corrosion and regulable structure/composition/functionalities. Actually, C_*x*_N_*y*_ based catalysts coupled with a cocatalyst, such as Ni_2_P, have displayed some activities/selectivity/stability for yielding valuable chemicals/fuels *via* upcycling the monomers (*e.g.*, ethylene glycol) released by pre-treating the polyesters (PET and PLA) at room temperature and under anaerobic conditions. Interestingly, C_*x*_N_*y*_ based photocatalysts, *e.g.*, C_3_N_4_, even exhibit some activity/selectivity/robustness for photocatalytic upcycling of untreated PS into value-added chemicals at raised temperature, in organic solvent (acetonitrile) and in air. But the efficiencies of non-metal C_*x*_N_*y*_ based photocatalysts are still much lower compared to those of metal-based photocatalysts, making their industrial utilization impossible at the moment. Compared with the above single-photon-absorber based photocatalysts, composite photocatalysts comprising two or even more photo-absorbers are also summarized in this review. They are categorized into inorganic based composite photocatalysts and inorganic/organic based composite photocatalysts. Only one inorganic composite photocatalyst is discussed in this review. The Pt cocatalyst loaded CdO_*x*_/CdS/SiC (Pt-CdO_*x*_/CdS/SiC) photocatalyst exhibits low H_2_ evolution activities from upcycling the untreated PE at raised temperature, in concentrated alkaline solution and under anaerobic conditions. These are mainly attributed to the strong C–C/C–H bonds of inert PE and lack of strongly oxidative ROS, *e.g.*, ˙OH radicals, owing to the unfavourable valence band edge potentials of CdS or SiC together with anaerobic reaction conditions. For inorganic/organic composite photocatalysts, two MOF based composites, Ag_2_O/Fe-MOF and ZnO/UiO66-NH_2_, are introduced in this review. Ag_2_O/Fe-MOF exhibits good H_2_ evolution activities for upcycling the PEG/PET/PE MPs. Especially, Ag_2_O/Fe-MOF shows some activity in upcycling PEG MPs into acetic acid. Interestingly, ZnO/UiO66-NH_2_ shows good efficiencies for upcycling untreated PLA or PVC into acetic acid with outstanding selectivity accompanied by some H_2_ evolution, in water, at room temperature and in air. The good efficiencies of both Ag_2_O/Fe-MOF and ZnO/UiO66-NH_2_ are mainly attributed to the rational synthesis strategy to yield Ag_2_O or ZnO NPs encapsulated in the pores of a MOF structure, which ensures strong interaction between Ag_2_O or ZnO NPs and the MOF structure for rapid charge separation/transfer, as well as excellent scattering of ultra-little Ag_2_O or ZnO NPs exposed with a high surface area. Another interesting study about inorganic/organic composite photocatalysts reports the synthesis of poly-oxalate combined polymeric C_3_N_4_, *i.e.*, V-substituted phosphomolybdic acid clusters/g-C_3_N_4_ nanosheets (VPOM/CNNS), for upcycling untreated PE/PEG/PP/PVC/PAA in water or organic solvent (acetonitrile) to generate a value-added chemical (formic acid) at room temperature and in an O_2_ atmosphere. This activity arises from the strong oxidation ability on the photo-induced holes from VPOM and the efficient Z-scheme charge transfer in VPOM/CNNS. Currently, although some advancements have been realized in this field, tremendous challenges are required to be overcome in the future, which is anticipated to bring about numerous opportunities in this field. For instance, the realistic application of photocatalytic plastic upcycling is severely restricted by the insufficient activity, selectivity and stability together with the low cost-effectiveness of photocatalysts. First, the inefficient use of the whole solar spectrum seriously restricts the maximum efficiency for solar-to-chemical conversion using the photocatalysis technique. Second, the high recombination rate of photo-excited electrons and holes in photocatalysts further limits the efficiency of photocatalysts seriously. This mainly arises from the different time scales for the generation/recombination of electron–hole pairs (picosecond to nanosecond) and surface redox reactions (millisecond to second). Third, the easy aggregation of photocatalyst powder suspended in aqueous solution significantly impedes the performance of powder-form photocatalysts for large-scale applications. Besides, recycling use of these photocatalysts *via* regeneration/re-activation is also challenging, since the separation of these powder-form photocatalysts from the suspension reaction system is difficult. Lastly, the long-term use of photocatalysts will lead to the gradual reduction of performance due to the reduced crystallinity and covered active sites by the product. Thus, herein, we summarize these challenges and possible opportunities in the following three sections:

### 4.1 Regulation of the characteristics of photocatalysts for increased activity/selectivity/stability

(1) Currently, no studies report photocatalysts with atomic-scale active sites in this field. So catalysts with atomic-scale reactive sites, such as single atoms, bi-single atoms and clusters, can be screened and developed for photocatalytic upcycling of various types of plastics (*e.g.*, PE, PP, PVC, PS and PET) into targeted value-added chemicals/fuels with excellent activity/selectivity/stability.

(2) Only limited engineering methods, *e.g.*, loading cocatalysts, element doping, morphology controlling and constructing Z-scheme/type II junctions, have been applied in this field. Thus, those advanced engineering routes of photocatalysts, *e.g.*, phase engineering, defect engineering, facet engineering and band structure engineering, can also be utilized for photocatalytic plastic upcycling.

(3) The cocatalyst plays a significant role in enhancing the activity/selectivity/stability of the photocatalyst. But currently only a few studies report the loading of cocatalysts (*e.g.*, Pt NPs, Ni_2_P NPs, MoS_2_ and NiMo) for photocatalytic plastic upcycling. And no insightful studies on the functional mechanism of these cocatalysts have been performed and reported. So more studies can be focused on engineering the composition/structure of the cocatalyst and its interaction with photocatalysts for tuning their activity/selectivity/stability for specific upcycling reactions.

(4) Currently, all the metal sulphide based photocatalysts reported in this field are based on Cd-based photocatalysts and suffer from notorious toxicity in realistic applications. Thus, Cd-free metal sulphide based photocatalysts can be screened and developed for photocatalytic plastic upcycling.

(5) Certain photocatalysts, *e.g.*, metal sulphides/selenides/phosphides, usually suffer from inferior photo-/thermal-/chemical-stability, compared to those of metal oxides. Their stability can be enhanced by the following strategies: (a) combining with other photocatalysts/co-catalysts (*e.g.*, metal oxides and metals) to boost electron–hole separation/transfer with reductive/oxidative electron/hole transfer to other photocatalysts/co-catalysts for avoiding self-reduction/-oxidation; (b) coating with a metal oxide layer to avoid chemical corrosion from the acidic/alkaline reaction environment.

(6) Cheap and robust C_*x*_N_*y*_ catalysts can be studied more owing to their unique advantages of earth-abundance, strong absorption of light and suitable oxidation abilities.

### 4.2 Advanced characterization and theoretical computations for revealing the structure–activity relationship and insightful reaction mechanisms

(1) A variety of *in situ* characterization techniques, such as *in situ* XPS, *in situ* ESR, *in situ* FTIR, *in situ* Raman, *in situ* AFM-KPFM, *in situ* XAS, *in situ* TAS, *in situ* SPV and *in situ* TSPL, can be utilized to reveal the structure–activity relationship and reaction mechanism, especially the *in situ* time-resolved characterization to study the femtosecond-scale kinetics of electron/hole separation/transfer/trapping/recombination in catalysts.

(2) It still remain unknown how the ROS is involved in photocatalytic plastic upcycling reactions. Various *in situ* characterization techniques, especially *in situ* ESR and *in situ* FTIR, can contribute to the study of ROS involved reactions, in which inert and untreated polyester/polyolefins can be efficiently upcycled into valuable short-chain chemicals/fuels.

(3) Online GC-TCD/FID and HPLC systems can be established and utilized to track and monitor the intermediates and products in photocatalytic plastic upcycling for revealing the insightful reaction mechanism under realistic conditions.

(4) Based on experimental results, theoretical computations, especially operando computation approaches, can be utilized to gain further insights into the structure–performance correlation in photocatalysts for plastic upcycling. They can also be applied to study the reaction mechanism *via* revealing the reaction thermodynamics/kinetics in plastic upcycling.

### 4.3 Advanced technologies for the realistic application in this area

(1) Currently, most of the photocatalytic reactions were conducted at 25 °C. The infrared region of the solar spectrum should be utilized to raise the reaction temperature for enhancing the reaction rates. Inexpensive and scalable reactors should be developed, which can efficiently utilize infrared light to raise the temperature of the reaction system and reserve the heat inside the reactor to keep the reaction system at a desirable temperature without external heating. Thus, the rates of photocatalysis reactions can be enhanced greatly.

(2) Seawater can be utilized to upcycle these plastic wastes to avoid the use of limited fresh water resources.

(3) Flow reactors can be utilized to avoid the overoxidation of chemicals to form tremendous CO_2_ generated in photocatalytic upcycling.

(4) In photocatalytic plastic upcycling, abundant CO_2_ might be generated due to the overoxidation of plastics, especially when an air atmosphere is applied. Thus, CO_2_ concentration should be monitored in photocatalytic plastic upcycling. And the efficient capture of the yielded CO_2_ and its further conversion into value-added carbon-based chemicals using identical photocatalysts should be studied.

(5) Earth-abundant and cheap cocatalysts can be developed to significantly increase the rate, selectivity and stability of photocatalysts for large-scale and cost-effective plastic upcycling using sunlight. Especially, efficient, low-cost and scalable loading techniques should be explored to atomically disperse these highly effective cocatalysts onto photocatalysts.

(6) Studies should be more focused on one-step photocatalytic upcycling of plastics without any pre-treatment.

(7) Currently, most reactions are conducted in aqueous solution. However, it is very hard for plastics to be suspended well in aqueous solution. More research should be conducted in some organic phase solvent (*e.g.*, acetonitrile and dichloromethane) to better suspend and/or dissolve plastics and ensure better interaction between the catalyst and reactant/intermediate together with more rapid mass transfer.

(8) Currently, all the photocatalytic plastic upcycling is conducted based on one reactor system, which cannot meet the requirements for realistic applications. Reaction systems containing multiple reactors with photocatalysts possessing different functions can be designed and constructed. For examples, one reactor containing metal oxide photocatalysts can be used to cleave the C–C/C–O/C–N/C–H bonds of plastics and yield monomers/oligomers/small molecules. Furthermore, these yielded monomers/oligomers/small molecules can be further transferred to another reactor containing metal sulphides/C_*x*_N_*y*_, which possess mild oxidation abilities to transform these chemicals to acquire value-added chemicals without over-oxidizing them to yield CO_2_.

(9) A solar simulator (AM 1.5G, 100 mW cm^−2^) is utilized in most reactions for photocatalytic plastic upcycling. For realistic applications in the future, solar concentrators can be applied to increase the photon intensity to achieve significantly increased efficiency.

(10) More efficient and cost-effective pre-treatment strategies can be developed and adopted to be combined with the photocatalysis technique for catalytic upcycling of plastics into value-added chemicals/fuels *via* environmentally benign and cost-effective routes.

(11) In realistic applications, it is very challenging to separate plastics and many of them are mixed with each other. Thus, more studies on photocatalytic upcycling of mixed plastics should be conducted to accelerate the development of realistic plastic upcycling techniques.

## Author contributions

S.-Z. Q. conceived the topic and structure of this manuscript. J. R. wrote the major part of the manuscript. A. T.-K. summarized the table, wrote some part of the manuscript and helped revise the manuscript. All the other authors have helped revise the manuscript.

## Conflicts of interest

There are no conflicts to declare.

## Supplementary Material
